# Morphological and transcriptomic analyses of stem cell-derived cortical neurons reveal mechanisms underlying synaptic dysfunction in schizophrenia

**DOI:** 10.1186/s13073-023-01203-5

**Published:** 2023-07-28

**Authors:** Annie Kathuria, Kara Lopez-Lengowski, Bradley Watmuff, Rakesh Karmacharya

**Affiliations:** 1 Harvard University, MGH Center for Genomic Medicine, Massachusetts General Hospital, 185 Cambridge Street, CPZN6, Boston, MA 02114 USA; 2 Chemical Biology Program, Broad Institute of MIT & Harvard, Cambridge, MA USA; 3 Department of Psychiatry, Harvard Medical School, Boston, MA USA; 4 Program in Neuroscience, Harvard University, Cambridge, MA USA; 5 Schizophrenia & Bipolar Disorder Program, McLean Hospital, Belmont, MA USA; 6 Program in Chemical Biology, Harvard University, Cambridge, MA USA; 7 Harvard Stem Cell Institute, Cambridge, MA USA

**Keywords:** iPSC, Dendritic spines, Schizophrenia, Neurexin, Clozapine

## Abstract

**Background:**

Postmortem studies in schizophrenia consistently show reduced dendritic spines in the cerebral cortex but the mechanistic underpinnings of these deficits remain unknown. Recent genome-wide association studies and exome sequencing investigations implicate synaptic genes and processes in the disease biology of schizophrenia.

**Methods:**

We generated human cortical pyramidal neurons by differentiating iPSCs of seven schizophrenia patients and seven healthy subjects, quantified dendritic spines and synapses in different cortical neuron subtypes, and carried out transcriptomic studies to identify differentially regulated genes and aberrant cellular processes in schizophrenia.

**Results:**

Cortical neurons expressing layer III marker CUX1, but not those expressing layer V marker CTIP2, showed significant reduction in dendritic spine density in schizophrenia, mirroring findings in postmortem studies. Transcriptomic experiments in iPSC-derived cortical neurons showed that differentially expressed genes in schizophrenia were enriched for genes implicated in schizophrenia in genome-wide association and exome sequencing studies. Moreover, most of the differentially expressed genes implicated in schizophrenia genetic studies had lower expression levels in schizophrenia cortical neurons. Network analysis of differentially expressed genes led to identification of NRXN3 as a hub gene, and follow-up experiments showed specific reduction of the NRXN3 204 isoform in schizophrenia neurons. Furthermore, overexpression of the NRXN3 204 isoform in schizophrenia neurons rescued the spine and synapse deficits in the cortical neurons while knockdown of NRXN3 204 in healthy neurons phenocopied spine and synapse deficits seen in schizophrenia cortical neurons. The antipsychotic clozapine increased expression of the NRXN3 204 isoform in schizophrenia cortical neurons and rescued the spine and synapse density deficits.

**Conclusions:**

Taken together, our findings in iPSC-derived cortical neurons recapitulate cell type-specific findings in postmortem studies in schizophrenia and have led to the identification of a specific isoform of NRXN3 that modulates synaptic deficits in schizophrenia neurons.

**Supplementary Information:**

The online version contains supplementary material available at 10.1186/s13073-023-01203-5.

## Background

Schizophrenia is a chronic, debilitating psychiatric disorder that affects ~ 1% of the population globally and is characterized by hallucinations, delusions, disordered thought processes, and cognitive deficits [1, 2]. Convergent evidence from genetic studies, imaging studies, and postmortem investigations point to a central role for synaptic dysfunction in the neurobiology of schizophrenia [3–6]. There were early indications from postmortem and neuroimaging investigations that showed evidence for differences in brain structure in schizophrenia that arise from reduction in dendrites and synapses without loss of neurons or glial cells [7, 8]. Examination of brains from schizophrenia patients in the 1980s had described significant reduction in the volume of the gray matter and the cortex, accompanied by increased ventricle size [9]. Structural magnetic resonance imaging (MRI) studies also confirmed the reduction in gray matter volume and increase in ventricle size in schizophrenia [10, 11]. MRI studies further showed the presence of thinning in schizophrenia in a number or cortical areas, including in first-episode psychosis patients [12, 13]. Recent studies using positron emission tomography have shown significant reductions in levels of synaptic vesicle glycoprotein 2A (SV2A) in cortical areas, but not in the hippocampus [4]. Detailed postmortem human studies in schizophrenia consistently show abnormalities in dendritic spines and synapses in specific neurons in the cerebral cortex [14, 15]. Brains from schizophrenia patients show reduction in dendritic spine density in layer III, but not layer V, pyramidal neurons in the cerebral cortex [16–18]. The lower dendritic spine density in these cortical pyramidal neurons in postmortem brains correlates with a reduced number of thin plastic spines in schizophrenia, even though the number of larger mature spines remains unchanged [19].

One confounding factor in interpreting neuronal differences in postmortem brains is the many years of illness and treatment prior to death. It is unclear whether the postmortem findings reflect intrinsic features of the underlying disease biology that leads to the disorder or whether they arise from disease progression, epigenetic effects, or antipsychotic treatment over decades. In addition, it is not clear whether the lower dendritic spine density in cortical neurons in schizophrenia arise due to innate deficits in neuronal biology or if they arise due to increased synaptic pruning later in life [20]. Since GWAS and de novo mutations show that many genetic differences in schizophrenia converge on synaptic biology, we hypothesized that synaptic deficits observed in postmortem brains may reflect the intrinsic complex genetic risk in schizophrenia which can be examined in cortical pyramidal neurons differentiated from induced pluripotent stem cells (iPSCs) [3, 21]. In this study, we differentiated iPSCs from seven patients with schizophrenia as well as seven healthy control subjects to generate cortical neuron cultures that include specific cortical pyramidal neuron subtypes of interest in the disease biology of schizophrenia. We report on our investigation of those neurons using morphological analyses of dendritic spines and synaptic puncta, functional studies using calcium imaging and RNA-seq experiments to identify genes and pathways that were differentially expressed in schizophrenia cortical neurons. Our results demonstrate that iPSC-derived cortical neurons recapitulate cell type-specific synaptic deficits described in postmortem brain studies in schizophrenia and point to specific biological pathways that may be involved in modulating the dendritic spine and synapse deficits in schizophrenia.

## Methods

### Differentiation of iPSCs to cortical pyramidal neurons

With approval from the Institutional Review Board (IRB) at Massachusetts General Hospital and McLean Hospital, schizophrenia and healthy subjects were recruited to obtain fibroblasts and reprogram them into iPSCs, as described previously [22, 23]. An independent experienced team at McLean Hospital recruited and screened patients, obtained informed consent, collected detailed clinical histories, and isolated fibroblasts through skin punch biopsies. The subject enrollment process included a Structured Clinical Interview for DSM Disorders (SCID), a standard research instrument for ascertaining diagnoses for research studies, as well as detailed examination of their clinical records to corroborate the diagnoses and history of treatment response. Healthy individual with no history of psychiatric diagnoses or treatment, matched for age and sex, were recruited as control subjects. We undertook power calculation to find the sample size based on the effect size in postmortem brain studies and in studies of dendritic spines in rodent neuronal cultures. Demographic information on the subjects had been described previously and are also included in Additional file 1: Table S1 [22–24]. The cohort studied included seven Caucasian subjects diagnosed with schizophrenia and treated at McLean Hospital. The average age of the subjects at the time of recruitment was 44 years, with an age range of 23–67 years, and included four male subjects and three female subjects. Seven Caucasian healthy control subjects that were included in the study had an average age of 43.3 at the time of recruitment, with an age range of 24–55 years, and included three male subjects and four female subjects. We validated the reprogrammed iPSCs sequentially through examination of human embryonic stem cell-like morphology, karyotype analysis, expression of pluripotency markers through immunocytochemistry, and evidence of trilineage potential (Additional File 2) [23]. To differentiate iPSCs to cortical pyramidal neurons, iPSC cultures were maintained in NutriStem hPSC XF medium (Reprocell, 01–0005) until they reached confluency and were transferred to plates coated with Geltrex (Thermo Fisher, A1413202) for neuronal differentiation, with a plating density of 1 million cells/well in a 6-well well dishes. During days 0–9 of neuronal differentiation, cells were maintained in a 50/50 mix of N2/B27 media containing half N2 medium (485 mL Neurobasal medium (Life Technologies, 21,103,049), 5 mL N2 (Gibco, 17,502,001), 5 mL Glutamax (Thermo Fisher, 35,050,061), 5 mL penicillin–streptomycin (Thermo Fisher 15,070,063), and half B27 medium (10 mL B27 Supplement (Gibco, 17,504,044), 480 mL DMEM medium (Sigma-Aldrich, D6421), 5 mL Glutamax, 5 mL penicillin–streptomycin) supplemented with 10 μM SB431542 (Sigma-Aldrich, S4317), 1 μM dorsomorphin (Sigma-Aldrich, P5499), and 100 nM LDN193189 (Sigma-Aldrich, SML0559) with daily media changes. After day 10, cultures were maintained in the N2/B27 media without the supplemental factors with daily media changes. On day 30, cultures were switched to BrainPhys neuronal medium (StemCell Technologies, 05790) with biweekly, half media changes.

### In vitro trilineage differentiation and pluripotency potential

The iPSC lines were differentiated to three germ layers with STEMdiff Trilineage Differentiation Kit (StemCell Technologies, 05230) (Additional file 2). The iPSCs were maintained in StemFlex medium (Thermo Fisher, A3349401) on matrigel plates. Upon confluency, iPSCs were dissociated with Versene (Thermo Fisher, 15,040,066) and plated at the following seeding densities (300,000 cells/well for both endoderm and ectoderm, 100,000 cells/well for mesoderm). Cells were fixed with 4% paraformaldehyde and stained for the following lineage-specific markers: endoderm: FoxA2 (Cell Signaling, 8186S, 1:400), Sox17 (Cell Signaling, 81778S, 1:1600); mesoderm: Brachyury (Cell Signaling, 81694S, 1:1600), NCAM (Cell Signaling, 3576S, 1:250); ectoderm: Nestin (Cell Signaling, 33475S, 1:1600), Pax6 (BioLegend, 901,301, 1:250). Undifferentiated iPSCs were assessed for pluripotency with the markers Nanog (Cell Signaling, 4893S, 1:2000) and Oct-4A (Cell Signaling, 2890S, 1:1600). Details on the antibodies used are listed in Additional file 1: Table S2.

### RNA extraction and qPCR

RNA was collected from cortical neurons differentiated from iPSCs for nine control and eight schizophrenia lines. Cells were lysed in 500 μl Trizol (Thermo Fisher, 15,596,026) and transferred to an Eppendorf tube. After 200 μl chloroform was added, the tube was inverted 3 times and spun for 20 min at 14,000* g* at 4 °C. The aqueous layer containing RNA was extracted and further purified using the Qiagen RNeasy RNA mini kit and cDNA generated using the High-Capacity cDNA Reverse Transcription Kit (Applied Biosystems, 4,368,814). Pre-validated qPCR forward and reverse primers (Sigma-Millipore) were diluted to a final concentration of 1 µM forward and 1µM reverse (F/R mix). For each primer, a primer mix was prepared consisting 1uL F/R mix, 2µL 5 × HOT FIREPol EvaGreen qPCR Mix Plus (Mango Biotechnology, 08–25-00008), and 5µL RNase-free water. Two milliliter cDNA was added to each well of a 384 LightCycler 480 multiwell plate (Roche, 05102430001) followed by the primer mix. The qPCR was run on the Roche LightCycler 480 II with two technical duplicates. Details on the primers used are listed in Additional file 1: Table S3.

### Immunocytochemistry

Differentiated cortical neurons plated at a density of 25,000 cells/well on 24-well glass imaging plates were fixed at day 90. Cells were fixed in 4% paraformaldehyde for 30 min, washed for 5 min with PBS (phosphate-buffered saline) three times, and then permeabilized in PBST (PBS + Tween). Cells were subsequently blocked in blocking buffer (PBS + 2.5% goat serum, 2.5% donkey serum) for 1 h at room temperature and then incubated with primary antibodies diluted in PBS + 1% goat serum and 1% donkey serum overnight at 4 °C. The primary antibodies were removed, and samples were rinsed for 5 min with PBS three times. The cells were then incubated with secondary antibodies diluted in PBS + 1% goat serum and 1% donkey serum for 1 h at room temperature. Cells were rinsed again for 5 min with PBS three times and stored in PBS in the presence of ProLong Gold antifade mountant (Thermo Fisher, P36934). Details on the primary and secondary antibodies used are listed in Additional file 1: Table S2.

### Calcium imaging in cortical neurons

Differentiated cortical neurons at day 60 of differentiation were plated and cultured on glass coverslips. On the day of imaging, neurons were incubated at a 37 °C in imaging buffer (HEPES buffer supplemented with bovine serum albumin (BSA) 0.3% w/v and 1 μM tetrodotoxin (Abcam, AB120054)) containing 5 µM Fluo-4 AM (Thermo Fisher, F-14201) for 30 min. The cells were washed with imaging buffer and coverslips were placed into a chamber slide containing warm imaging buffer. Cells were recorded on a Leica confocal microscope at × 20 for 60 s before and after applying 30 mM KCl. The fluorescence intensity of twenty cell bodies selected from each field of view was measured over the course of the experiment using a defined region of interest in ImageJ. The fluorescence intensity for each cell was normalized to F_0_, the fluorescence intensity of the cell at time = 0 s. The maximum F/F_0_ values after exposure to KCl were compared between control and schizophrenia lines.

### Western blot

Samples were obtained from lysed cells and protein concentration measured using a Pierce BCA protein assay kit (Thermo Fisher, 23,225). Ten micrograms of protein extract for each sample was run on a Criterion TGX Precast gel 4–20% (Bio-Rad, 5,671,094). Gels were then transferred to Immobilon-P Transfer Membrane (Millipore, IPVH00010, pore size: 0.45 um, PVDF) for 90 min at 100 V. The membrane was blocked for 1 h in Odyssey blocking buffer (Li-Cor, 927–40,000) and then incubated overnight with primary antibodies diluted in the blocking buffer at 4 °C. The membrane was washed for 5 min with TBST (tris-buffered saline, 0.1% Tween) three times and subsequently incubated with secondary antibody for 1 h diluted in Odyssey blocking buffer. Blots were scanned on the Li-Cor Odyssey Clx Imaging System and analyzed using ImageJ. Details on the antibodies used are listed in Additional file 1: Table S1.

### Transcriptome analysis

The RNA-seq library was constructed using the Illumina RiboZero TruSeq Stranded Total RNA Library Prep Kit (Illumina) and the 100 nt, paired-end configuration sequencing was performed using the Illumina NovaSeq6000 platform. Sixty million reads were obtained for each sample on average. For gene expression analysis, reads were trimmed with Cutadapt and subsequently aligned to the reference genome (hg38 UCSC assembly) using TopHat v2.0.14 and Bowtie v2.10 with default parameters and RefSeq annotation (genome-build GRCh38.p9) [25]. The distribution of alignments was analyzed using Cufflinks v2.2.1 and FPKM (fragments per kilobase of exon model per million reads mapped) values were quantile normalized. Differential expression testing was performed with Cuffdiff v2.2.1 [26]. As per the guidelines for the limma package, we converted the FPKM values into values to a log2 scale (y = log2(FPKM + 0.1) and processed the samples using standard R packages [27]. The contrast between SCZ vs CON was designed such that a positive fold change referred to upregulation of the gene in SCZ. Statistical significance of the events was evaluated based on the raw *p*-value cutoff, since no genes survived the Benjamini–Hochberg adjustment at 5% FDR. The raw data for this has been uploaded in NCBI and DEG analysis is provided as Additional file 3.

### Gene ontology analyses

Gene ontology (GO) was done using MetaCore + MetaDrug™ version 19.1 build 69,600 for process, localization, and molecular function. Reactome, KEGG, and Hallmark analysis was used on all differentially regulated genes with the Functional Enrichment Analysis unit of HOMER v.3 [28]. The genes shown in the figures are ones that reached significance (*p<*0.05).

### Automated image analysis

To avoid any potential biases in analyzing data, an automated analysis protocol was used to analyze the images of dendritic spines and synapses. Cells were fixed and stained antibodies raised against neuronal marker MAP2, postsynaptic marker Homer, and presynaptic marker Bassoon. Quantitative image analyses of cortical neuron cultures were conducted using Opera Phenix at × 63 magnification using Harmony high-content analysis software 4.9 (PerkinElmer). Spine density and synaptic puncta density from 125 randomly selected fields were quantified. Automated quantification of seven CON and SCZ lines was performed with three replicates each. Using the Harmony® high-content analysis software 4.9 (PerkinElmer), cells positive for nuclei with CUX1/CTIP2 were identified, using channel selection with absorption wavelength of 358 nm (ultraviolet) and emission wavelength at 461 nm (blue). Once the nuclei were identified, MAP2 staining of the neurites was highlighted (Additional file 1: Fig. S1). Using the SER (saddle, edges, ridges) feature methods, the image analysis “building block” analyzed the intensity structure of the defined image region for the occurrence of typical patterns. The cell body was excluded based on filter property feature defined by size, and the new population was defined as neurites. Once the neurites were identified and de-convoluted, the spines and synaptic puncta were identified using the “find spots” image analysis pipeline, selecting neurites as the population of interest (Additional file 1: Fig. S2). Spines were selected via size and fluorescence intensity. For identification of synaptic puncta, the “identify population” feature was used to filter the image by property for puncta positive for both Homer and Bassoon, selected based on their emission wavelength at 488 and 568 respectively (Additional file 1: Fig. S2).

### NRXN3 knockdown

For the knockdown experiments, purified NRXN3 204 GFP shRNA lentiviral particles (GeneScript LPP-CS-HSH1466L-LVRU6GP-01–100) targeting custom 522 bp region without targeting 7 RefSeq protein-coding variants for human NRXN3, in psi-LVRU6GP with U6 promoter, eGFP, puromycin (10^8 TUs/mL) or scrambled GFP lentiviral particles were transduced in cortical neurons from a control iPSC line at day 85 of differentiation. Knockdown was verified using RT-PCR to confirm that the transcript NRXN3 204 had no expression. GFP-positive cells were analyzed for spine/synaptic puncta quantification.

### NRXN3 overexpression

Overexpression of NRXN3 204 GFP lentiviral particles (purified lentiviral particles for custom NRXN3 204 (ATG + 522 bp) sequence in pReceiver-Lv122 with CMV promoter, C-eGFP tag, puromycin (8 TUs/mL), or scrambled GFP lentiviral particles) were transduced in cortical neurons from a SCZ iPSC line at day 85 of differentiation. Overexpression was verified using RT-PCR to confirm that the increased expression of the NRXN3 204 transcript. GFP-positive cells were analyzed for spine/synaptic puncta quantification.

### Clozapine experiments

Cortical neurons were treated with 10 µM clozapine for 24 h on day 89 of differentiation and then fixed and stained on day 90. The spines and synapses were quantified in an automated manner, pre-selecting only CUX1-positive cells for analysis. Automated quantification of spines and synapses were carried out in seven SCZ lines, with two replicates each.

### Experimental design and statistical analyses

We calculated our sample size to attain at least 95% power for the comparisons of mean dendritic spine density values between CON and SCZ. The calculations were based on effect sizes reported in postmortem studies of SCZ and CON and in vitro studies that examined the response to clozapine in rodent neurons [17, 29, 30]. Rodent neurons treated with clozapine had a robust increase in dendritic spine density, with a *p*-value < 0.001. The study showed that the ∆ for spine density means between clozapine treated vs. untreated samples was 2.14 spines/10 µm, with the standard error of the mean of ~ 0.4 for a sample size of 7 in each category. Analyses of dendritic spine and synaptic puncta were conducted using the Harmony® high-content analysis software 4.9 (PerkinElmer). ImageJ (NIH) was used for analyzing data from calcium imaging experiments. Data normality was tested using the Kolmogorov–Smirnov test. We used unpaired *t*-test with Welch’s correction as required for normal distribution. For non-normal distributions, Mann–Whitney *U* test was performed. Statistical analyses of the data and the graphs were conducted in GraphPad Prism 9.0 (GraphPad Software). For the transcriptomic data, differential expression testing was performed per the limma package guidelines in R [27]. Raw *p*-value cutoff of < 0.05 was used to determine significance and differences determined by sex were not considered. The number of data points (*n*) and the statistical significance (*p* value) are stated in the figure legends.

## Results

### Dendritic spine density in specific cortical neuron subtypes

**Fig. 1:**
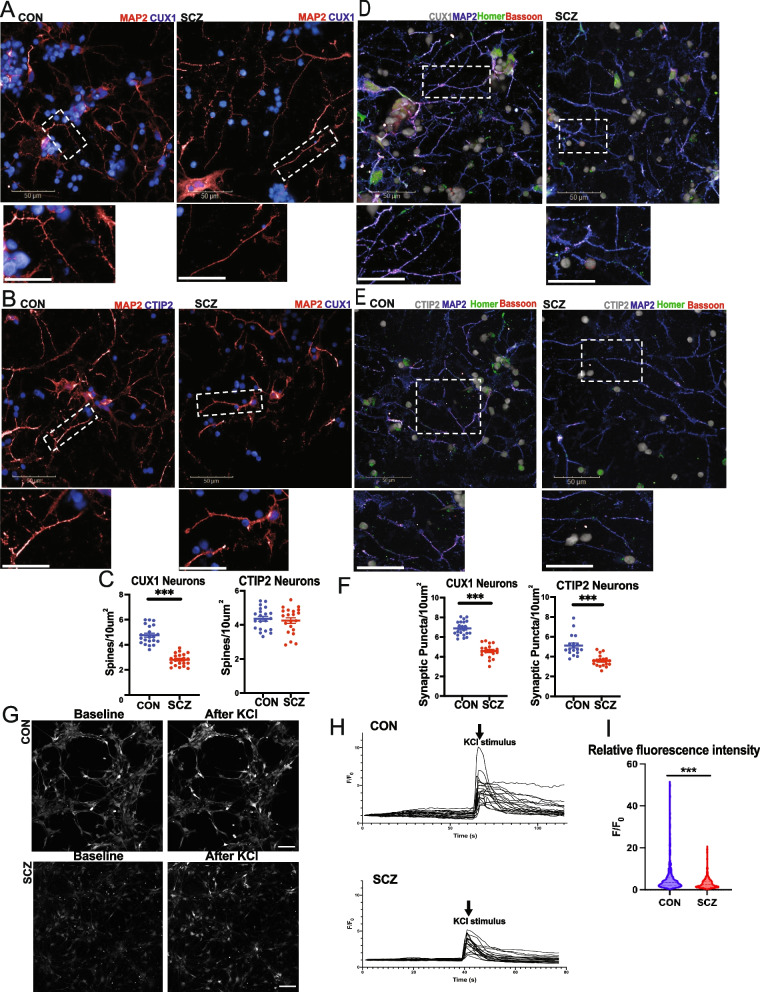
Dendritic spines and synapses in SCZ and CON cortical neurons. **A**,**B** Images of dendritic spines in SCZ and CON neurons, labeled with layer III marker CUX1 or layer V marker CTIP2 and MAP2—arrows point to representative spines. **C** Automated quantification of spine density in dendrites in CUX1 or CTIP2 neurons from seven CON and seven SCZ lines, with three replicates each (box and whisker plot min to max, *p* < 0.001, unpaired *t*-test with Welch’s correction). **D,E** Images of synaptic puncta in SCZ and CON neurons, labeled with layer III marker CUX1 or layer V marker CTIP2, MAP2, presynaptic marker Bassoon (red) and postsynaptic marker Homer (green)—arrows point to representative synaptic puncta with overlap of Bassoon and Homer. **F** Automated quantification of synaptic puncta in neurons from seven CON and seven SCZ lines. SCZ neurons showed significantly fewer synapses compared for both CUX1 and CTIP2 neurons (dot plots, mean + / − SEM, *p* < 0.001, unpaired *t*-test with Welch’s correction). Scale bar = 20μm. **G** Images of Ca^++^ imaging in CON and SCZ neurons before and after 30 mM KCl. Scale bar = 100μm. **H** Trace of Ca.^++^ fluorophore intensity before and after KCl. **I** Change in fluorescence intensity in cell bodies upon KCl exposure. SCZ neurons had lower increase in fluorescence intensity after KCl (mean + / − SEM, *p* < 0.001, Mann–Whitney *U* test)

We reprogrammed fibroblasts from schizophrenia patients and healthy control subjects to generate iPSC lines using established reprogramming methods, as we have described previously [22, 23]. We utilized dual SMAD inhibition to generate cortical neurons from seven schizophrenia and seven control iPSC lines in order to compare dendritic spines and synapses between the two groups [31]. After differentiating iPSCs to generate 3-month-old cortical neuron cultures, we utilized automated high-content imaging analysis to quantify the neurite lengths as well as density of spines and synaptic puncta in dendrites connected to specific cortical neuron subtypes, using layer-specific cortical pyramidal neuron markers [32] (Additional file 1: Fig. S3-S4). In neurons expressing the cortical layer III marker CUX1, there was a significant reduction in dendritic spine density in neurons from schizophrenia iPSCs compared to neurons from healthy control iPSCs (Fig. 1A–C). However, there was no significant difference in dendritic spine density between the schizophrenia and control groups in cortical neurons expressing the layer V marker CTIP2 (Fig. 1A–C). This cell type-specific difference in dendritic spine density is consistent with postmortem findings of dendritic spine reductions specifically in cortical layer III neurons in schizophrenia [16–18].

While the postmortem findings arose from investigations of patient brains after decades of illness and treatment, it is intriguing that our results in iPSC-derived neuron patients recapitulate the postmortem findings of subtype-specific deficits in cortical neurons in schizophrenia. This suggests that the complex genetic risk underlying schizophrenia results in intrinsic deficits in forming dendritic spines in specific neuronal subtypes. Given the observed differences in dendritic spines, we hypothesized that the overall number of synapses would also be reduced in schizophrenia. We quantified synaptic puncta in the cortical neurons with automated high-content imaging analysis by counting the overlap of presynaptic (Bassoon) and postsynaptic (Homer) puncta and found reduced synaptic puncta density in cortical neurons from schizophrenia iPSCs, with a more pronounced reduction in synaptic puncta density in cortical neurons expressing CUX1 (Fig. 1D–F; Additional file 1: Fig. S5).

### Functional studies of cortical neurons with Ca^2+^ imaging

We examined whether these synaptic differences in schizophrenia cortical neurons were accompanied by differences in functional activity. We carried out live-cell calcium imaging of the cortical neurons generated from schizophrenia and healthy control iPSCs in the setting of depolarization with KCl, using an approach we have described previously [33]. We found that while there was no difference in spontaneous calcium transients between schizophrenia and healthy control neurons at baseline, schizophrenia cortical neurons showed significant reduction in depolarization-related neuronal firing when compared to neuronal firing patterns in healthy control neurons (Fig. 1G–I, Additional file 1: Fig. S6).

### Transcriptomic profiles of iPSC-derived cortical neurons

**Fig. 2:**
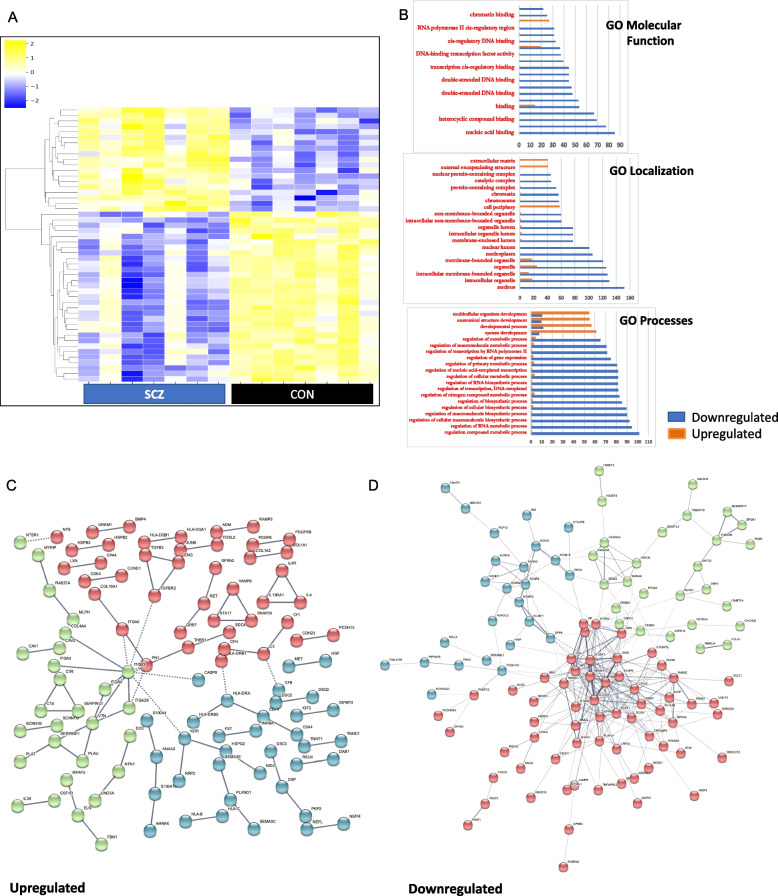
Transcriptomic analyses of SCZ and CON cortical neurons. **A** Heatmap of the top 50 DEGs for seven CON and seven SCZ neurons. **B** GO analysis showing the top 20 categories in molecular function, localization and processes. **C**,**D** Network map of genes upregulated and downregulated genes in SCZ generated using STRING software. PPI enrichment *p*-value < 1.0e − 16 for both networks. Each node represents all proteins produced by a single, protein-coding gene locus. Red colored nodes are query proteins and first shell of interactors. Green nodes indicate secondary interactions. Blue nodes represent tertiary interactions. Edges represent protein–protein associations indicating that proteins jointly contribute to a shared function, though they do not necessarily interact with each other physically. Line thickness is proportional to edge confidence

The presence of morphological differences as well as functional deficits in schizophrenia cortical neurons prompted us to interrogate biological mechanisms that underlie these deficits using RNA-seq experiments. We undertook transcriptomic experiments using RNA isolated from 90-day-old cortical neurons differentiated from seven schizophrenia iPSC lines and compared them to cortical neurons differentiated from seven control iPSC lines [34]. The heatmap from RNA-seq data of the top 50 differentially expressed genes (DEGs) showed a clear pattern between the schizophrenia and healthy control groups (Fig. 2A; Additional file 1: Fig. S7). Using a raw *p*-value < 0.05, we found 2223 genes that were upregulated and 2514 genes that were downregulated in the schizophrenia neurons. We compared these DEGs with the most recent list of common and rare variant genes implicated in schizophrenia in GWAS and exome sequencing studies (Table 1) [35, 36]. Of the 106 prioritized protein-coding genes that were implicated in schizophrenia in the GWAS, we found that expression of 17 of these genes was significantly different in the schizophrenia cortical neurons. Of the 30 rare coding variant genes that were significant at FDR < 0.05 in the schizophrenia exome sequencing study, expression of 9 of these genes was significantly different in the schizophrenia cortical neurons [36]. Taken together, 18% of the genes implicated in schizophrenia in the GWAS and exome sequencing studies had significant differences in expression in the schizophrenia cortical neurons. The expression patterns of DEGs that were implicated in the schizophrenia genetic studies were mostly downregulated in the schizophrenia cortical neurons—80% of these genes had significantly lower levels of expression in schizophrenia cortical neurons compared to healthy neurons while only 20% of these genes had higher levels of expression in schizophrenia cortical neurons (Table 1).

**Table 1:** Schizophrenia GWAS and rare coding variant genes in DEGs. Rare coding variants are listed in red. SP4 was in implicated in SCZ in both GWAS and exome sequencing studies

Gene	Log fold change	*p*-value	Expression in SCZ
STAG1	− 0.65432192	0.000211006	Downregulated
ASH1L	− 0.574034203	0.001639272	Downregulated
MSI2	− 0.628298006	0.002623759	Downregulated
FAM114A2	− 0.379608028	0.004309934	Downregulated
SP4	− 0.741815974	0.005321399	Downregulated
ZNF823	− 0.441175662	0.005530187	Downregulated
ZNF136	− 0.474551229	0.008610404	Downregulated
SF3B1	− 0.276285598	0.008889232	Downregulated
DCLK3	1.756895082	0.009724435	Upregulated
EIF2S3	0.29021066	0.010107875	Upregulated
ENOX1	− 0.885135386	0.012875532	Downregulated
MAD1L1	− 0.359021879	0.013981546	Downregulated
RB1CC1	− 0.485352017	0.016544659	Downregulated
BCL11B	− 1.536911061	0.022580399	Downregulated
WSCD2	− 1.422044855	0.024713595	Downregulated
SLC39A8	0.909995356	0.03063367	Upregulated
EMX1	1.63706111	0.03109794	Upregulated
RERE	− 0.765833586	0.031917862	Downregulated
HIST1H1E	− 0.914444985	0.033835341	Downregulated
NEGR1	− 1.11617521	0.033886208	Downregulated
DCC	1.286228948	0.039496096	Upregulated
PCDHA8	− 0.608954035	0.042243822	Downregulated
ANKRD12	− 0.560118184	0.046496813	Downregulated
HERC1	− 0.402594143	0.048675911	Downregulated

**Fig. 3:**
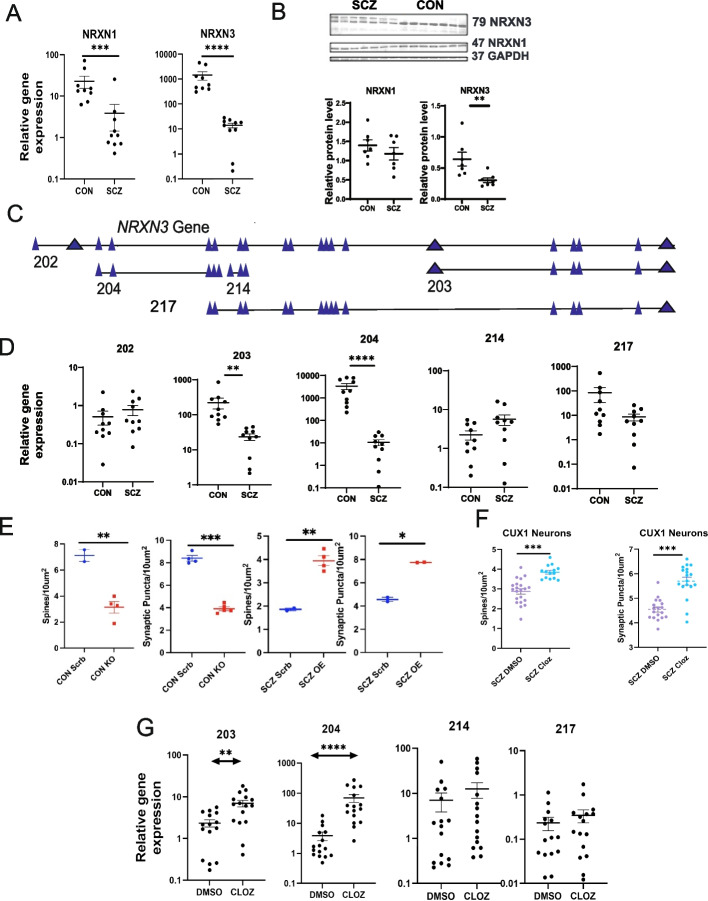
NRXN3 isoforms regulate density of dendritic spines and synapses in SCZ neurons. **A** SCZ neurons showed significant reduction (mean + / − SEM, Mann–Whitney *U* test) in mRNA expression of NRXN3 (*p* < 0.001) and NRXN1 (*p* = 0.0010). **B** Protein levels for NRXN3β and NRXN1 in seven SCZ and seven CON cortical neurons showed lower levels NRXN3β in SCZ (mean + / − SEM, Mann–Whitney *U* test, *p* = 0.0041) but no significant difference in NRXN1. **C** Annotation of NRXN3 isoforms, adapted from Ensembl. **D** Relative expression levels of NRXN3 isoforms—NRXN3 202 represents full gene. SCZ neurons showed significant reduction (mean + / − SEM, Mann–Whitney *U* test) of NRXN3 isoforms 203 (*p* < 0.001) and 204 (*p* < 0.001), but not in isoforms 202, 214, or 217. **E** Knockdown of NRXN3 204 in CON neurons resulted in reduction in dendritic spines (*p* = 0.0086) and synaptic puncta (*p* < 0.001) compared to scrambled control shRNA (mean + / − SD, unpaired *t*-test with Welch’s correction). Overexpression of NRXN3 204 in SCZ neurons resulted in increased dendritic spines (*p* = 0.0014) and synaptic puncta (*p* = 0.0364) compared to overexpression of a scrambled control (mean + / − SD, unpaired *t*-test with Welch’s correction). **F** CUX1 neurons from 7 SCZ lines treated with 10 μM clozapine in triplicates showed increased density of dendritic spines and synaptic puncta (dot plots with mean + / − SEM, *p* < 0.0001, unpaired *t*-test with Welch’s correction). **G** SCZ cortical neurons exposed to 10 μM clozapine for 24 h showed a significant increase (mean + / − SEM, Mann–Whitney *U* test) in NRXN3 203 (*p* < 0.0012) and 204 (*p* < 0.0001), but not of 214 or 217

We carried out gene ontology (GO) analyses of the DEGs, which showed significant downregulation in categories related to metabolic processes, biosynthetic processes, and non-membrane-bound organelles in schizophrenia, along with upregulation in extracellular matrix gene categories (Fig. 2B). Pathway analysis of upregulated genes showed a prominent role for collagen-related genes (Fig. 2C), while pathway analysis of downregulated genes revealed NRXN1 and NRXN3 as the central network hubs (Fig. 2D). We confirmed expression levels of the major DEGs from RNA-seq data using RT-PCR and western blot analyses. NRXN1 and NRXN3 expression was reduced in schizophrenia cortical neurons, and the NRXN3 reduction was the most robust and consistent between the different lines (Fig. 3A). Western blots showed significant reduction in NRXN3β protein levels in schizophrenia cortical neurons, but the NRXN1 protein levels were not significantly different between the schizophrenia and healthy control groups (Fig. 3B). These experiments showed a consistent and significant decrease in NRXN3 at the mRNA and protein level in cortical neurons from schizophrenia patients.

### Identification of NRXN3 isoforms

Neurexins comprise a family of membrane proteins that localize in synapses, modulate dendritic spine plasticity, and undergo alternative splicing to generate a large number of isoforms [37, 38]. We employed the Ensembl genome browser and UniProt to collate protein-coding isoforms of NRXN3 (202, 203, 204, 214, 217) that contain the laminin G-like binding domain, which is the binding site for neurexophilins (NXPHs) (Fig. 3C) [39]. RT-PCR experiments showed significant reduction in expression of the 203 and 204 isoforms in schizophrenia cortical neurons, with isoform 204 showing the most significant reduction (Fig. 3D). To gain insight into the relationship between this NRXN3 isoform and the identified synaptic differences, we used shRNA to selectively knockdown the NXRN3 204 isoform in cortical neurons from healthy control iPSCs (Additional file 1: Fig. S8). Knockdown of the NXRN3 204 isoform resulted in significant reduction in dendritic spine and synaptic puncta density in the healthy control cortical neurons, which phenocopied our findings in the schizophrenia cortical neurons (Fig. 3E,F). Conversely, we undertook experiments for overexpression of the NXRN3 204 isoform in schizophrenia cortical neurons using a lentiviral vector (Additional file 1: Fig. S8), which resulted in significant increase in dendritic spine and synaptic puncta density in the schizophrenia cortical neurons (Fig. 3E,F). Taken together, these results suggest a central role for the NRXN3 204 isoform in mediating synaptic differences in schizophrenia.

### Effect of clozapine on synaptic deficits

We investigated whether antipsychotic medications used to treat schizophrenia had any effects on the synaptic differences we had identified in the schizophrenia iPSC-derived cortical neurons. Clozapine is the most efficacious medication for the treatment of schizophrenia and it modulates a number of different cellular pathways relevant to schizophrenia [40–42]. Exposure to 10 μM clozapine for 24 h led to significant increase in dendritic spine and synaptic puncta density (Fig. 3G) in CUX1-positive cortical neurons from schizophrenia iPSC lines, partially rescuing the cell type-specific deficits that we had described above in schizophrenia (Fig. 1C,F). We found that clozapine also increased dendritic spine and synaptic puncta density in CUX1-positive cortical neurons from the healthy control iPSCs as well (Additional file 1: Fig. S9). We also examined whether clozapine modulates the aberrant expression of NRXN3 isoforms in schizophrenia. In cortical neurons from schizophrenia lines, exposure to 10 μM clozapine for 24 h resulted in increased expression of the NRXN3 203 and 204 isoforms, but not of the 214 and 217 isoforms (Fig. 3H). Taken together with the NRXN3 overexpression data (Fig. 3E,F), these results suggest that clozapine ameliorates synaptic deficits in schizophrenia cortical neurons by increasing expression of NRXN3 204.

## Discussion

The development of novel and efficacious therapies for schizophrenia has been hampered by a dearth of our understanding of the cellular and molecular underpinnings in schizophrenia and related psychotic disorders [43–46]. Somatic cell reprogramming methods enable in vitro investigations of different human neuronal cell types that are hypothesized to be involved in mediating the disease biology of psychiatric disorders such as schizophrenia [47–52]. Schizophrenia is highly heritable and genetic studies have led to the identification of nearly three hundred genetic risk loci for schizophrenia [35, 53]. While individual risk loci have very small effect sizes, recent GWAS and studies of de novo mutations show that many genetic differences in schizophrenia converge on synaptogenesis and synaptic function [3, 21, 54]. Recent studies for highly penetrant risk genes have led to intriguing new insights into cellular processes that may underlie the disease biology of schizophrenia [36, 55, 56]. The presence of common variants with small effect sizes makes it difficult to parse the roles of individual genes when the disease arises due to the combined effects of many different variants [57]. The ability to generate iPSCs from well-characterized schizophrenia patients and healthy subjects enables us to generate neuronal cells that have the same genetic background as the subjects and interrogate biological differences that result from the collective effect of the complex genetic backgrounds [58–62]. There is convergent data, including from iPSC-based studies, that implicate deficits in synaptic biology in the etiopathogenesis of schizophrenia [63–65]. Schizophrenia is typically diagnosed in adolescence or early adulthood [66]. This raises conceptual and technical limitations in using iPSC-derived neurons, which have transcriptomic profiles similar to those in fetal neurons [67–69]. However, our hypothesis considers the evidence that schizophrenia is a neurodevelopmental disorder with impairments in synaptic connectivity starting very early in life, though the full-blown psychotic episode only manifests in adolescence or early adulthood [70, 71]. In our study, we demonstrate that iPSC-derived cellular models can enable identification of innate biological differences in schizophrenia conferred by the underlying complex genetic risk.

There are well-replicated studies that show consistent abnormalities in dendritic spines and synapses in pyramidal neurons in different layers of the cerebral cortex [14, 72]. It has been hypothesized that modulation of dendritic spine density in cortical neurons may be a tractable approach for new therapeutic approaches in schizophrenia [73]. In brains from schizophrenia subjects, studies have reported significant reduction in dendritic spine density in layer III, but not layer V, cortical pyramidal neurons [16–18]. While cortical neurons generated from iPSCs in two-dimensional cultures do not have three-dimensional layered structures, the presence of the cells that typically reside in those layers can be distinguished by marker analysis [31]. In cortical neuron cultures differentiated from human iPSCs, the emergence of different layer-specific neurons follows the same temporal sequence as seen in the developing brain, with the neurons expressing layer VI markers appearing first, followed by stepwise emergence of pyramidal neurons expressing markers for the other layers [31]. By generating and examining different cortical neuron subtypes, we found that iPSC-derived cortical neurons that express layer III markers, but not those that express layer V markers, show significant differences in dendritic spine density in schizophrenia when compared to such neurons from healthy control subjects. One of the categories that the DEGs were enriched for were genes involved in the extracellular matrix, which is relevant given the important role that components of the extracellular matrix plays in the spine and synapse structure [74]. Recent studies with iPSC-derived neurons from schizophrenia subjects showed electrophysiological differences in a pattern that correlated with clinical features [61]. Our findings further show that the complex genetic risk in schizophrenia lends itself to specific cellular phenotypes and that iPSC-derived cortical neurons can recapitulate cell type-specific synaptic deficits described in postmortem brain studies.

The reason for the selective difference in dendritic spine density in layer III neurons remains to be understood. In the human brain, layer III is a major integration site for thalamocortical and corticocortical networks [75, 76]. As such, it has been suggested that the decreased dendritic spine density in layer III neurons in SCZ in postmortem brains may arise due to differences in inputs from the thalamus and other cortical areas [14, 77]. Along those lines, studies in animals show that decrease in constitutive NMDA receptor activity leads to reduction in cortical volume, accompanied by reduced spine density [78, 79]. Recent studies in human and primate cortices also show that dendritic spines in the middle laminar zone, compared to the deep laminar zone, receive a greater density of connections from both pyramidal neurons and interneurons [77]. Another hypothesis that has been described to account for decreased dendritic spines in the cortical pyramidal neurons is increased synaptic pruning by microglia [80]. However, the identification of these differences in dendritic spines in iPSC-derived neurons in vitro in monolayer cultures suggests that there may be intrinsic deficits that are cell autonomous. There are a few other differences between layer III neurons and layer V neurons that may be pertinent for the selective deficits in layer III neurons. One relates to the timing of its appearance in neurodevelopment. Layer V neurons appear earlier than layer III during normal brain development and in iPSC-derived cortical differentiation as well [81–83]. Another reason may relate to the possibility of aberrant mitochondrial function in layer III neurons in schizophrenia. Mitochondrial function is very important for the maintenance of dendritic arborization and deficits in mitochondrial transport to dendrites induced by inhibition of mitochondrial fission has been shown to result in reduction in spine density [84]. For example, impairing mitochondrial transport to dendrites by inhibiting mitochondrial fission decreases neuronal spine density [85–87]. The recent findings from other groups on mitochondrial deficits in schizophrenia iPSC-derived neurons may be relevant in this regard [48, 88–90]. Future investigations using single-cell transcriptomics may help elucidate the molecular landscape and mechanistic underpinnings for the selective differences in layer III neurons in schizophrenia.

Analyses of gene expression profiles in human postmortem brains show genes with enriched expression in the supragranular layers which are hypothesized to be involved in increased long-range projections in the cortex [91]. Studies have collated a human supragranular enriched set of 19 genes that comprise genes expressed in the upper layer of the cortex that are unique to the human brain relative to the mouse brain as well as genes that are expressed in layer V/VI in the mouse brain but had a shifted expression pattern to layer II/III in the human brain [91]. When we cross-referenced this human supragranular enriched set with the DEGs in our transcriptomic data, we found that two of these genes were upregulated in SCZ (COL6A1 and PRSS12) and two were downregulated in SCZ (BEND5 and C1QL2) (Additional file 3). COL6A1 encodes a type VI collagen that is expressed in the central nervous system [92] and our pathway analysis of the transcriptomic data had also shown a significant upregulation role for collagen-related genes (Fig. 2C). PRSS12 encodes motopsin, a serine protease that plays a role in neurodevelopment and cognitive function [93]. BEND5 encodes a mammalian transcriptional repressor involved in neurogenesis [94]. It is notable that one of the downregulated genes C1QL2 encodes a complement component that forms a complex with NXRN3 to regulate the function of postsynaptic kainate-type glutamate receptors [95].

Our findings lend support to the hypothesis, based on genetic and animal studies, that neurexins play a pivotal role in the disease biology of schizophrenia [96–98]. Studies in iPSC-derived neurons from schizophrenia patients with heterozygous NRXN1 deletions have shown specific deficits in neurotransmitter release and in synaptic function similar to those observed in engineered NRXN1-deficient neurons [99]. Neurexins have multiple isoforms and localize in synapses and are known to be involved in modulating dendritic spine plasticity [37]. After undertaking transcriptomic analyses of cortical neurons as well as knockdown and overexpression experiments, we identified the NRXN3 isoform 204 as a key NRXN3 isoform that mediates synaptic deficits in iPSC-derived cortical neurons in schizophrenia. Previous studies had described that transcriptomic organization at the isoform level show the most robust differences in disease specificity and effect sizes in neuropsychiatric disorders [100]. Alternative splicing of NRXN3 has been be described to induce trans-synaptic effects on synaptic function and plasticity in rodent studies [101]. Clozapine, the most effective medication for treatment of schizophrenia, had previously been described to modulate dendritic spine biology in rodent neurons in vivo and in vitro [29, 30]. We found that exposure to clozapine resulted in robust increases in dendritic spines and synapses in layer III cortical neurons and to increase levels of the NRXN3 204 isoform in the schizophrenia cortical neurons.

There are a number of caveats and limitations to our findings. Studying a specific iPSC-derived neuronal cell type in isolation will not fully capture the complexity of its functioning in the human brain. Since well-replicated postmortem studies had described significant reduction in SCZ in dendritic spines in cortical layer III, we had sought to generate these neuronal subtypes to investigate whether these postmortem differences are present in iPSC-derived neurons as well. The postmortem studies were undertaken in intact brains with a complex milieu that include cortical interneurons and glial cells. The contribution of glutamatergic and GABAergic to excitation-inhibition balance remain an active area of investigation and studies of human and primate cortices show evidence for the presence of dendritic spines that hare innervated by both other cortical pyramidal neurons and by interneurons [77, 102]. Dual SMAD inhibition does not result in appreciable numbers of GABAergic to quantify GABAergic synapses in these cultures [83]. Previously, when we studied interaction between excitatory and inhibitory neurons, we had differentiated excitatory and inhibitory neurons separately and co-cultured them [23]. We had found that GABAergic neurons had a disease-specific effect on synapse density in these co-cultures but we do not have information on whether these effects are specific to layer III neurons.

Over the last decade, several groups have been investigating the neurobiology underlying SCZ using iPSC-based disease modeling. While our studies focused on specific neuronal morphological features and differences in a specific synaptic protein, previous studies using iPSC-derived neurons have described differences in SCZ in several cellular pathways and processes, including glutamatergic neurotransmission, dopamine synthesis, Wnt signaling, synaptic connectivity, mitochondrial function, and oxidative stress [103–109]. These is crosstalk between these different pathways in the cellular context and they are integral to synaptic function. Many of these cellular pathways had also been hypothesized to play a role in SCZ based on genetic and postmortem studies [110–114]. Future iPSC-based studies can help delineate the interplay between these different cellular processes as they relate to the disease biology of schizophrenia.

## Conclusions

Our experiments with iPSC-derived cortical neurons from schizophrenia and healthy subjects led to the recapitulation of postmortem findings of cortical neuron subtype-specific dendritic spine deficits in schizophrenia, along with transcriptomic data showing primarily downregulation of genes implicated in schizophrenia genetic studies, and identification of a specific isoform of a synaptic protein that is involved in the cellular phenotype. The ability to model disease-related phenotype in vitro provides unprecedented new opportunities for the identification of novel therapeutic leads that can target the underlying neurobiological disease process in neuropsychiatric disorders.

## Supplementary Information

**Additional file 1.** Materials and Methods, List of antibodies, list of qPCR primers.

**Additional file 2.** Data of pluripotency of iPSC lines using trilineage differentiation.

**Additional file 3.** DEG analysis.

## Data Availability

The datasets generated during the current study have been deposited in NCBI’s Gene Expression Omnibus [115] are accessible through GEO Series accession number GSE164376 [34] (https://www.ncbi.nlm.nih.gov/geo/query/acc.cgi?acc=GSE164376). Analytical tools used in this study are mentioned in the “Methods” section with references.
