# Supplementary material for: Morphological and transcriptomic analyses of stem cell-derived cortical neurons reveal mechanisms underlying synaptic dysfunction in schizophrenia
Source: Genome Med. 2023 Jul 28;15:58. doi: 10.1186/s13073-023-01203-5 (PMC10375745; doi:10.1186/s13073-023-01203-5)
Supplement: Supplementary file 1 — Additional file 1. Materials and Methods, List of antibodies, list of qPCR primers. [file 13073_2023_1203_MOESM1_ESM.docx]

**Table S1: Demographic characteristic of cohort**

Healthy Control subjects

Average age: 43.3

Sex: 3 male, 4 female

Race: All 7 Caucasian

Schizophrenia subjects

Average age: 33

Sex: 4 male, 3 female

Race: All 7 Caucasian

**Table S2: List of antibodies.**

**Table S3: List of Primers**

| Primer | Forward Sequence (5'-3') | Reverse sequence (5'-3') |
| --- | --- | --- |
| CPLX2 | CCGAGATAAGTATTGGCTG | GATGCTCTCCTCTTCCTC |
| GABRA1 | ACTGTCTTTGGAGTAACAAC | CCATGCATAACCTCTCTTAG |
| GAD1 | CAATACCAACATGCCATCAG | ATTTAGGAGGAAGACCCTTG |
| NRXN1 | AGAACTGCATATACACCAGG | GATTCTTCAATGGCGATGTC |
| NRXN3 | CCAGAAATGTCTACTACTGTC | CATCTGATGTTGGCTGAATG |
| NRXN3 202 | AAGAACAGAGCCTAAATGCAC | AGCACACATACTCTGAGCC |
| NRXN3 203 | TGGAAAGGACAAAGGACGCC | ACTTCTCCAACCAGCCGAAC |
| NRXN3 204 | TTCTTACCACGACGGGCTAC | CATGAAGTTGTTGCTGACAGG |
| NRXN3 214 | GCTTACAATACCTGCTTGCC | ACAACTTTTCACTGTGCCC |
| NRXN3 217 | AACAACCCCTCCCTTCTAC | GCTGGGTATGTTTCCTCTTTG |
| NXPH1 | AACAAAGACTCAAAGAAGGC | GCACATGTGACCAAGTAG |
| RPL32 | GTGCAACAAATCTTACTGTG | CTGCCTACTCATTTTCTTCAC |
| SLC32A1 | AGAAGTCCTGGTCCATTATC | GATATTGATGACGAAGTGGG |
| SLC6A1 | CTCTTTCCACAACAATGTCTAC | GATGGAGAAGATGACGAATC |
| SYNPR | TGGTCTTTGGATTCTTGAAC | AAAGATATCTCTGTCCCGAAG |

Figure S1: Automated Spine Analysis

Figure S2: Automated Analysis Synaptic Punctas.

A

B

Figure S3: A. Representative images and reconstructions of neurites stained for MAP2. Scale bar: 20 μM. B. Quantification of mean primary neurite length quantified in 7 CON lines vs 7 SCZ lines. Mean±SEM. No significance is observed, tested via Unpaired t test with Welch's correction.

**
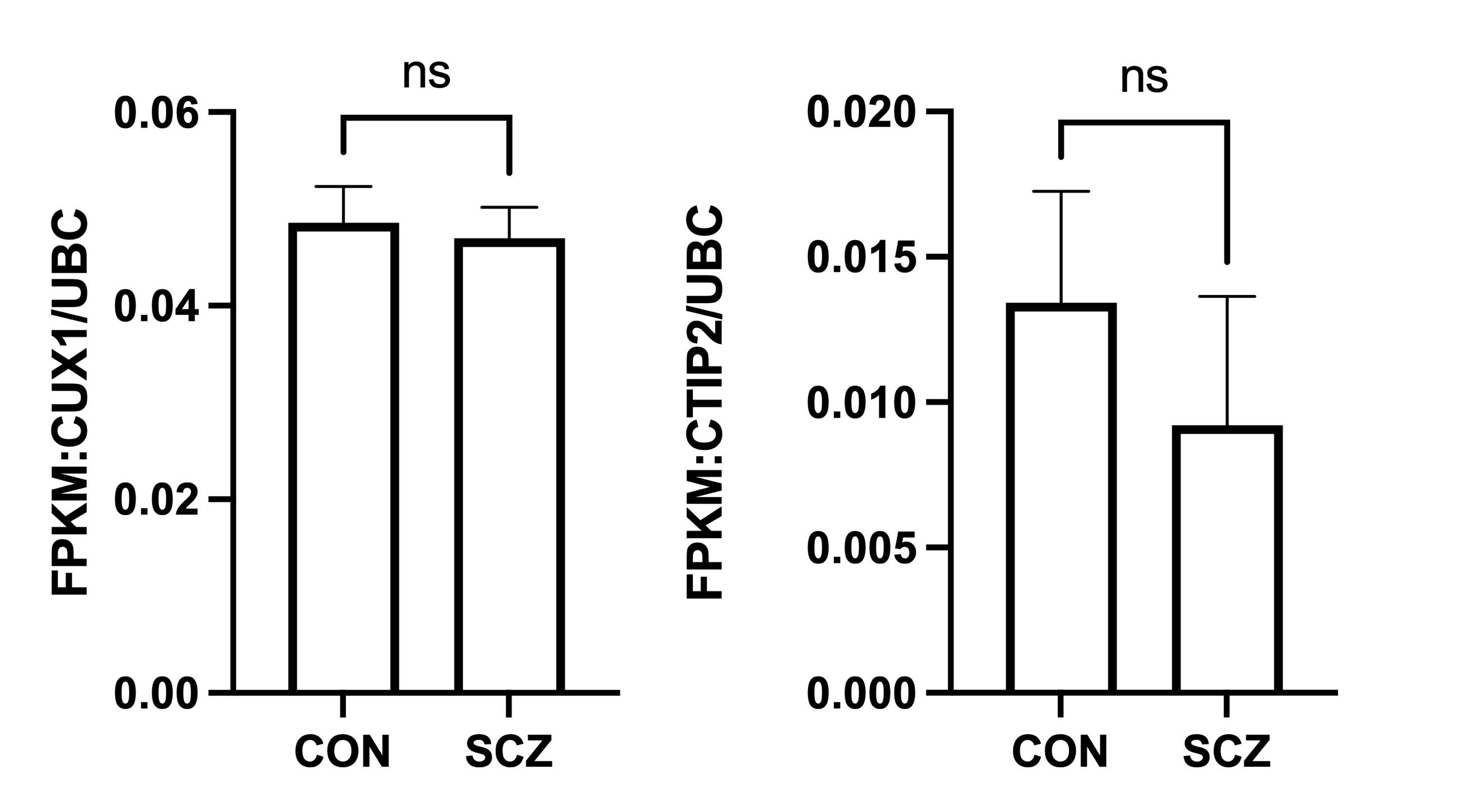
**

A.


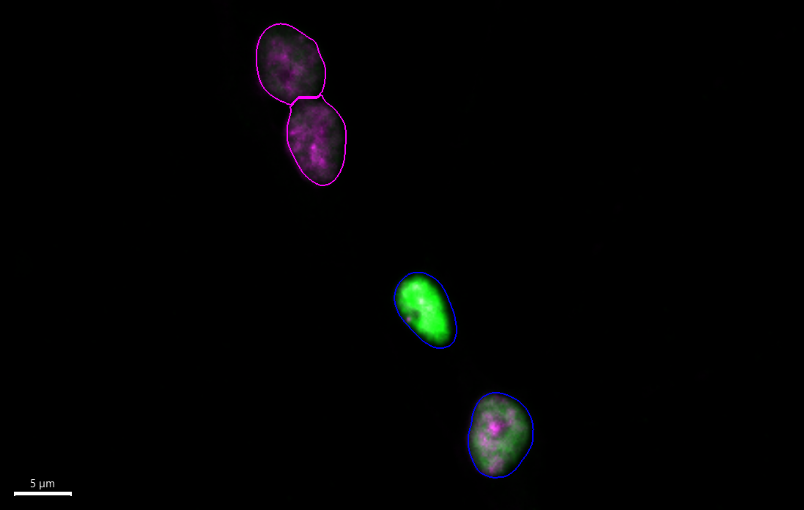
 **
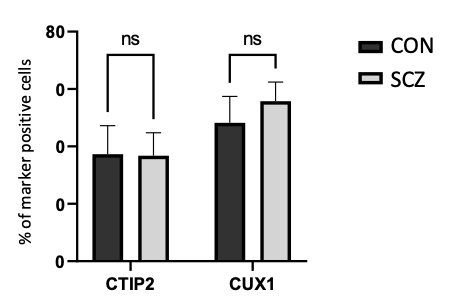
**

B.

Figure S4: A. Levels of gene expression for CUX1 and CTIP2 in day 90 neurons differentiated from 7 CON and 7 SCZ iPSC lines. Error bars show S.E.M. Data is N.S. (p>0.5).

B. Quantification of upper-layer and deep-layer neurons in SCZ and CON cortical neuron cultures. Day 90 cortical neuron cultures differentiated from CON and SCZ iPSCs were fixed and stained for cortical markers, CTIP2 (Green) and CUX1 (Magenta). Neurons were analyzed using Imaris and classified into two groups based on intensity: deep (CTIP2+; blue outline) and upper (CUX1+, CTIP2-; magenta outline) neurons. Scale bar is 5 µM. Percent positive is plotted over the total neuron count for each category. Data was collected from cortical neuron cultures for 6 different CON lines (n=6; 288 neurons total) and 4 different SCZ line (n=4, 253 neurons total) lines. Error bars show S.E.M. Data is N.S. (p>0.5).

Figure S5: Representative images of neuronal cultures used for quantification of synaptic puncta. Images for synaptic staining shown in the setting of knockdown NRXN3 in control neurons or overexpression of NRXN3 in schizophrenia neurons. Arrows point to representative puncta detected along the neurites. Scale bar: 20 μM.

**A.**

**B.**

Figure S6: A. Traces of baseline Ca^2+^ imaging patterns of CON and SCZ cortical neuron cultures under normal culture conditions before addition of KCl. B. Quantification of basal Ca^2+^ imaging data before addition of KCl. Error bars show S.E.M. Data is N.S. (p>0.5).

**
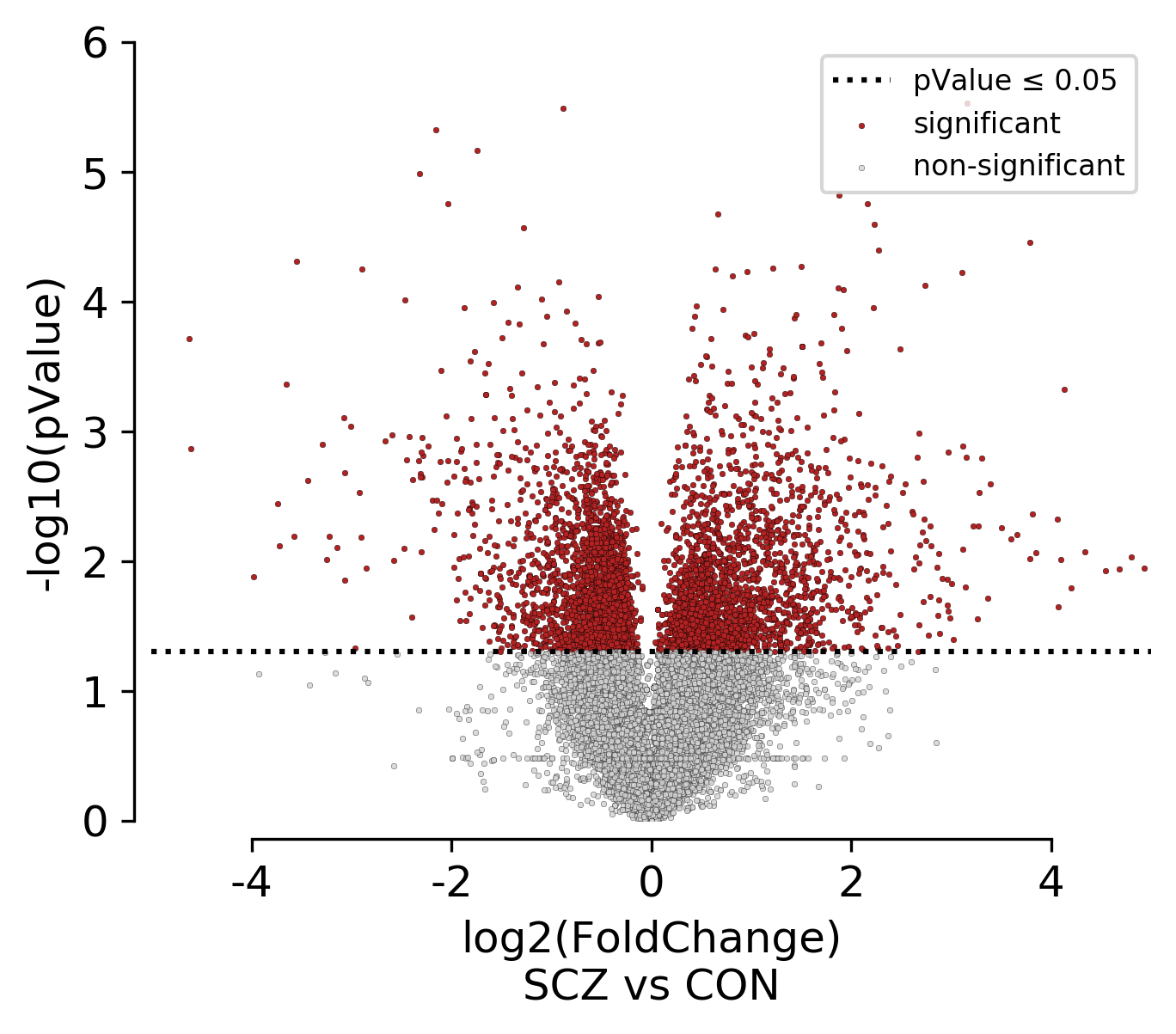
**

Figure S7: Volcano plot showing the DEGs. The top upregulated gene is TYRP1 while the top downregulated gene is CCK.

Figure S8: A. RT-PCR verification of NRXN3 204 isoform overexpression in a schizophrenia line. Relative expression of NRXN3 204 isoform following lentiviral transduction in a schizophrenia line. Data shown as MEAN±SEM, *p= 0.0286, Mann-Whitney test, normalization was checked. B. RT-PCR verification of NRXN3 204 isoform knockdown in a control line. Data shown as MEAN±SEM, **p=0.0097, Student t test with Welch’s correction.


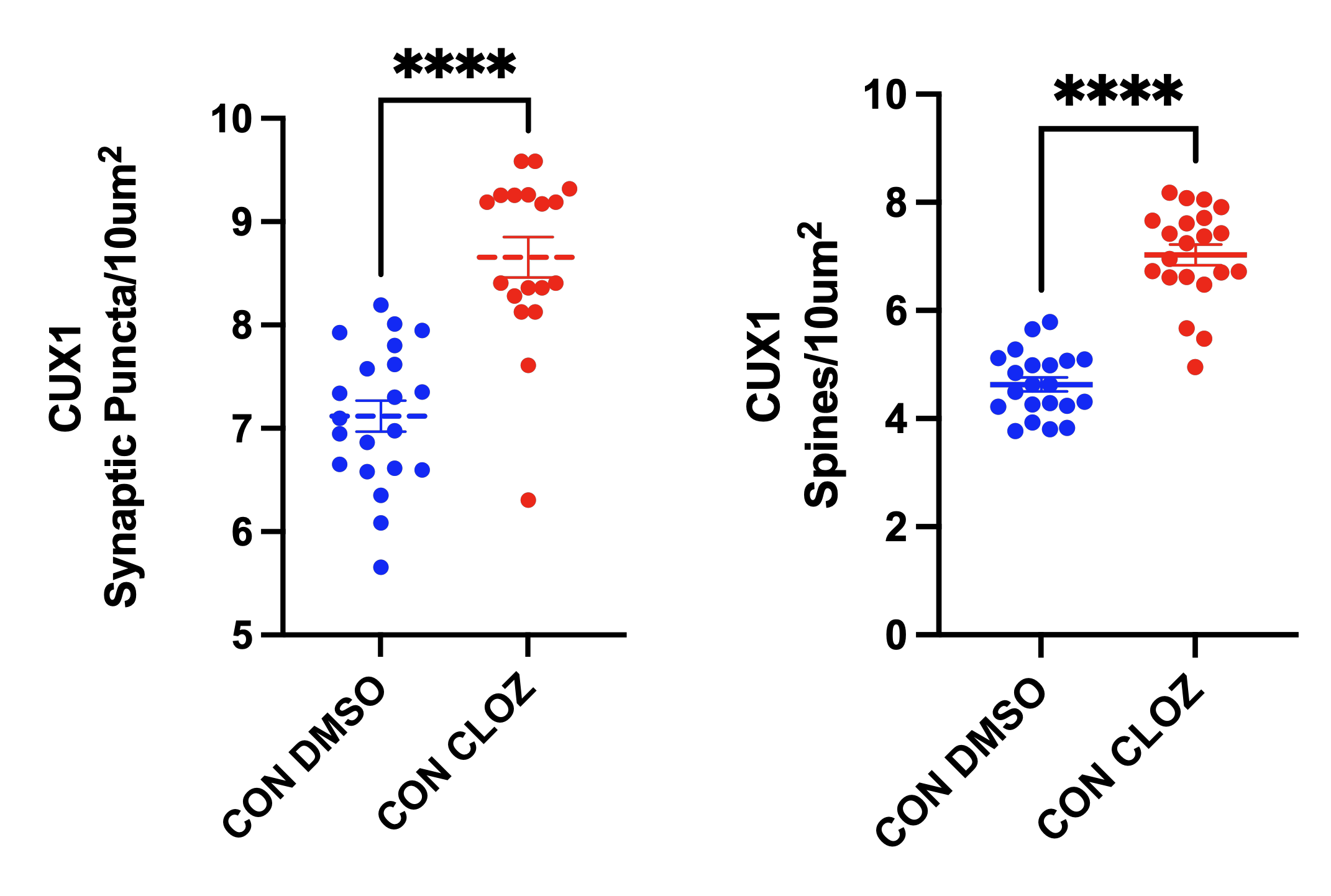


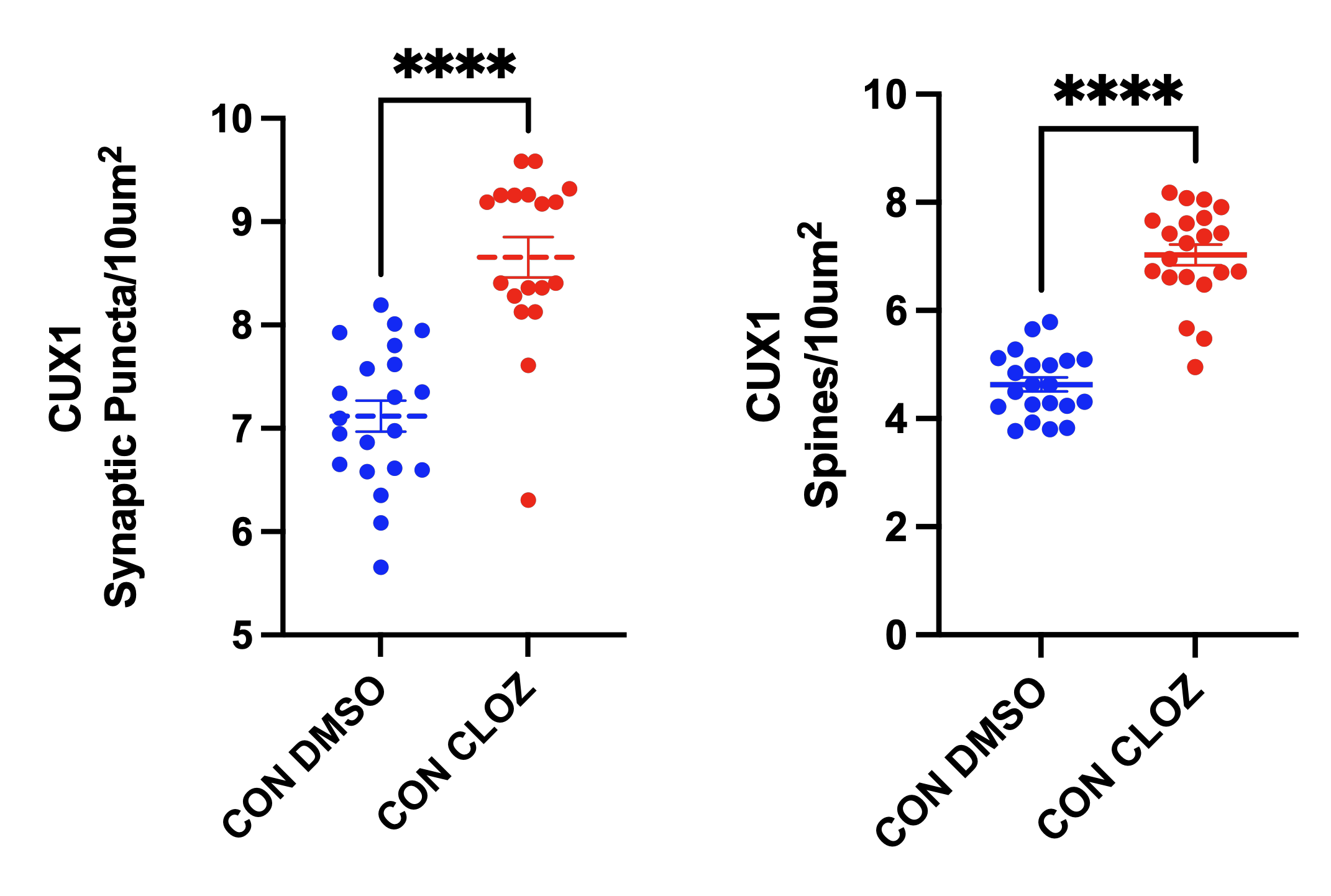


Figure S9: Quantification of dendritic spine density and synaptic puncta density in upper-layer CUX1 neurons differentiated from seven healthy control iPSC lines. Error bars show S.E.M. Unpaired t-test with Welch's correction with p<0.0001.
