# Supplementary material for: Morphological and transcriptomic analyses of stem cell-derived cortical neurons reveal mechanisms underlying synaptic dysfunction in schizophrenia
Source: Genome Med. 2023 Jul 28;15:58. doi: 10.1186/s13073-023-01203-5 (PMC10375745; doi:10.1186/s13073-023-01203-5)

ML 292
Control

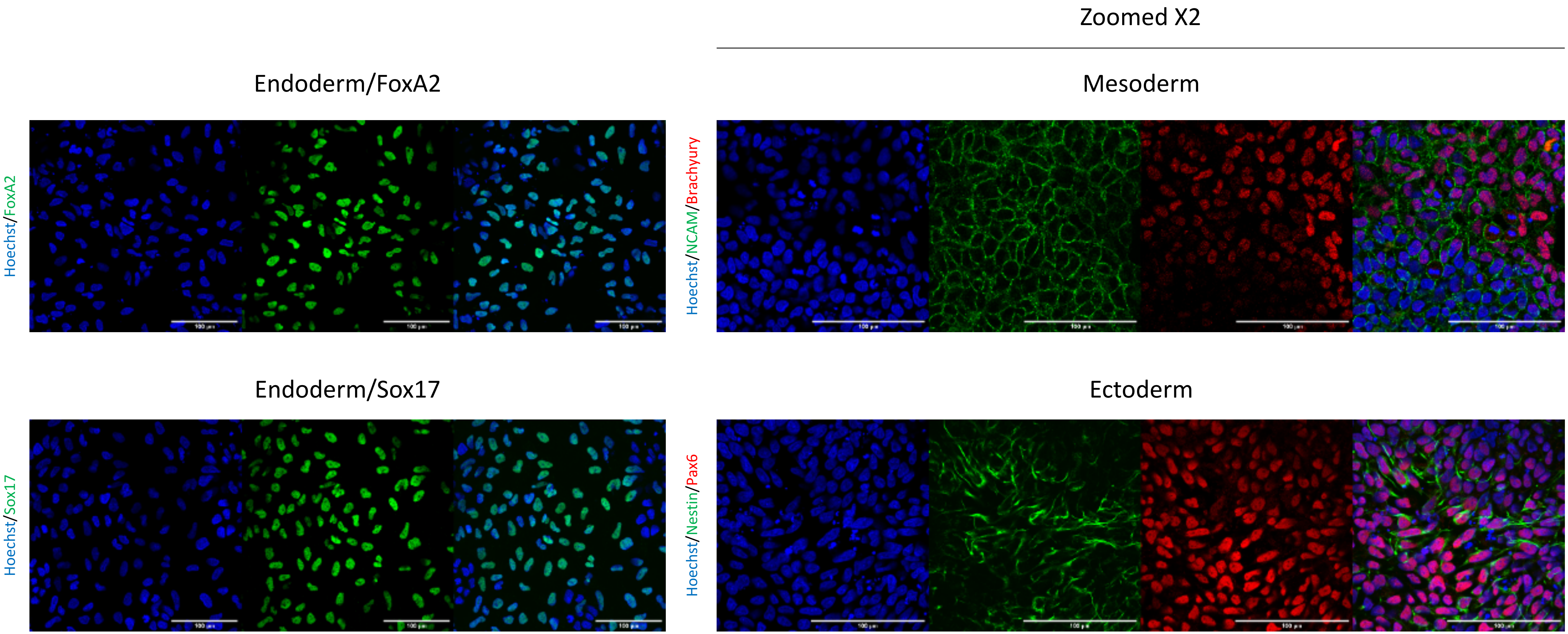

ML 226
Control

Zoomed X2

Endoderm/FoxA2

Hoechst/FoxA2

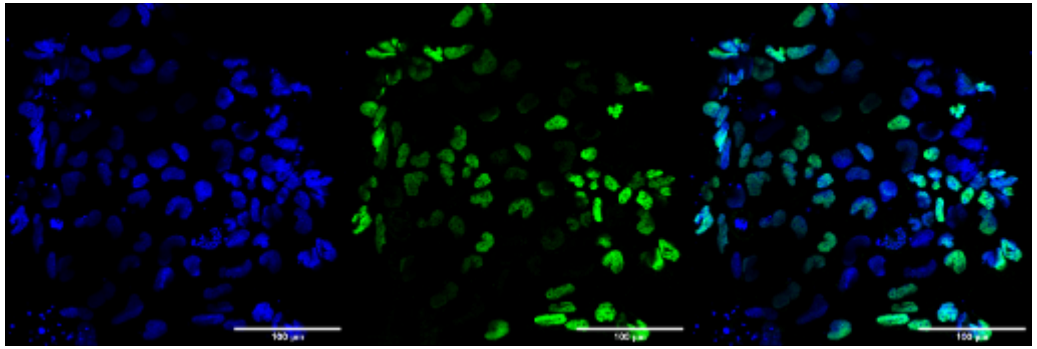

Mesoderm

Hoechst/NCAM/Brachyury

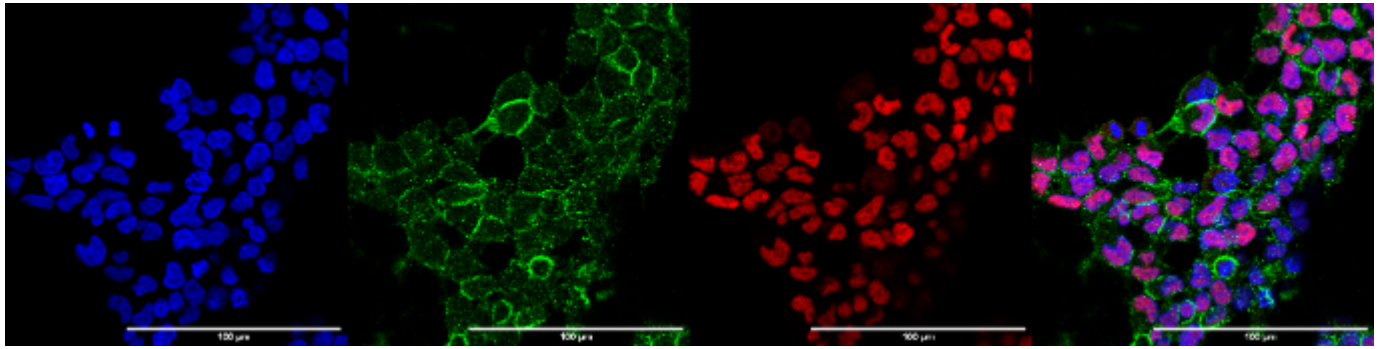

Endoderm/Sox17

Hoechst/Sox17

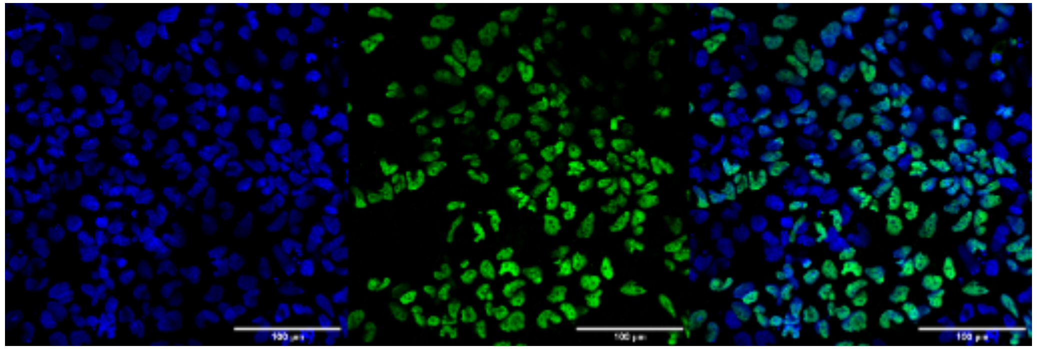

Ectoderm

Hoechst/Nestin/Pax6

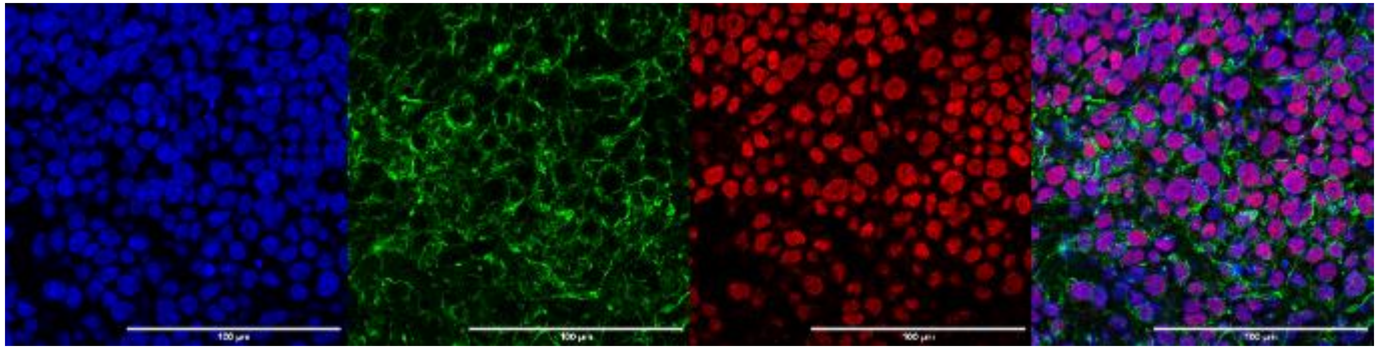

ML 126
Control

Zoomed X2

Endoderm/FoxA2

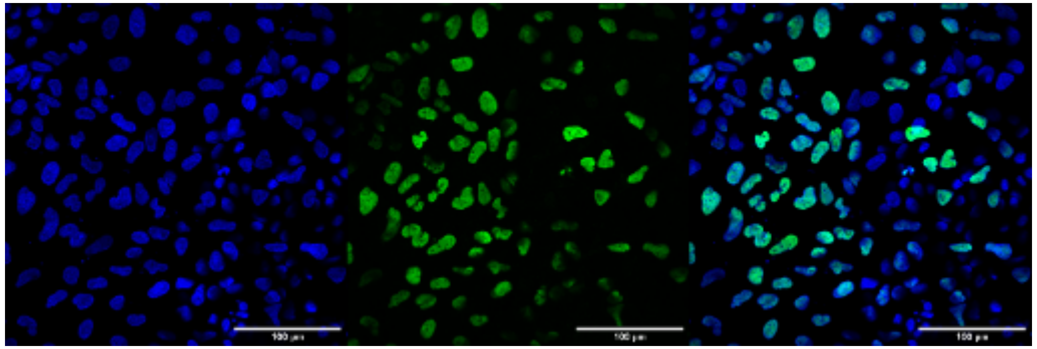

Mesoderm

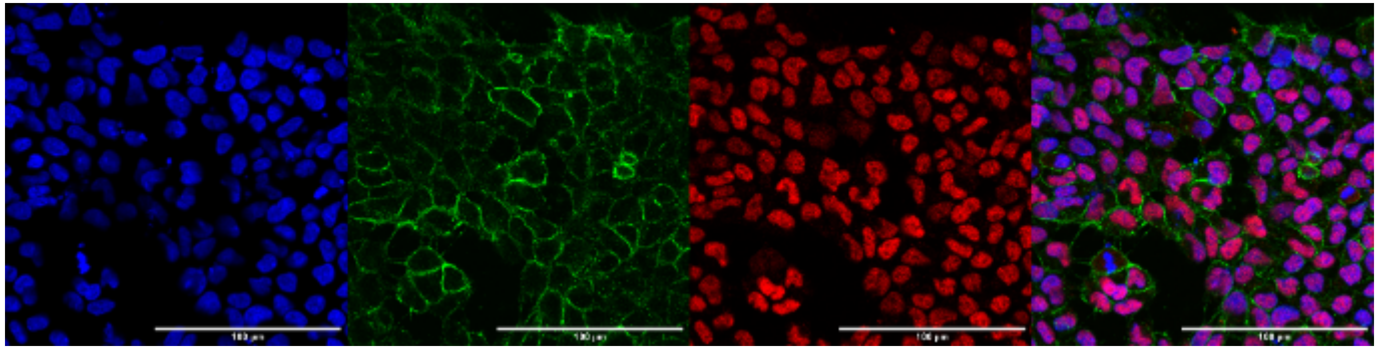

Endoderm/Sox17

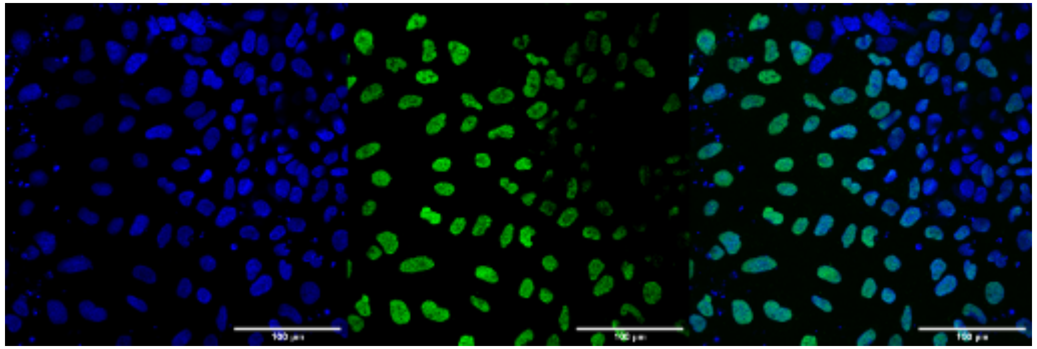

Ectoderm

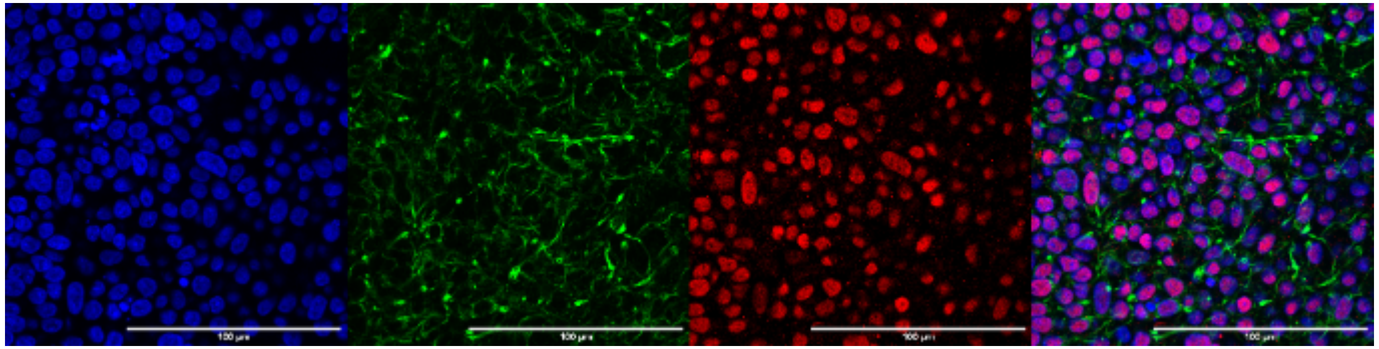

ML 135

Control

Zoomed X2

Endoderm/FoxA2

Hoechst/FoxA2

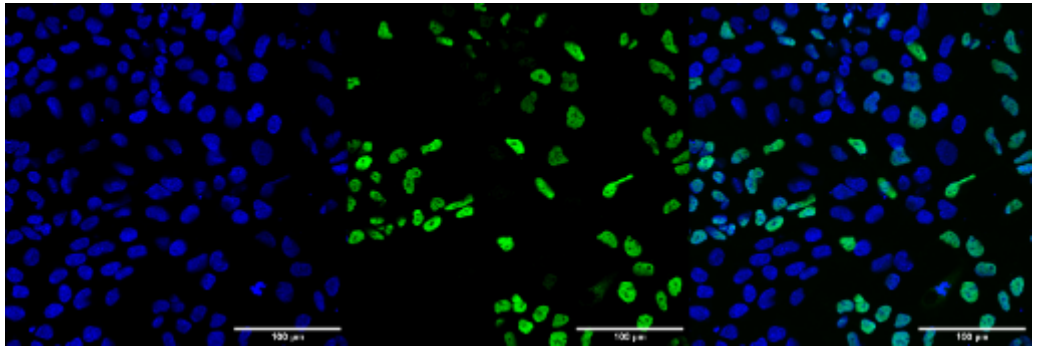

Mesoderm

Hoechst/NCAM/Brachyury

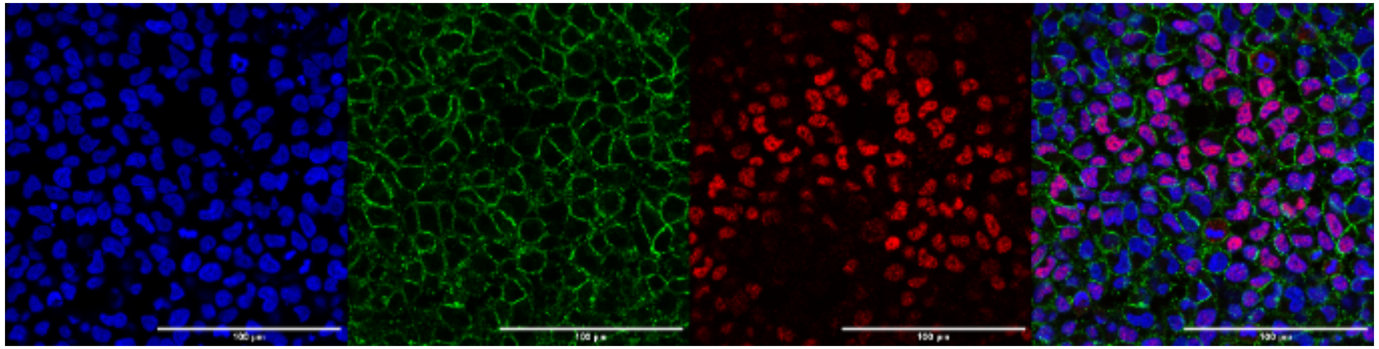

Endoderm/Sox17

Hoechst/Sox17

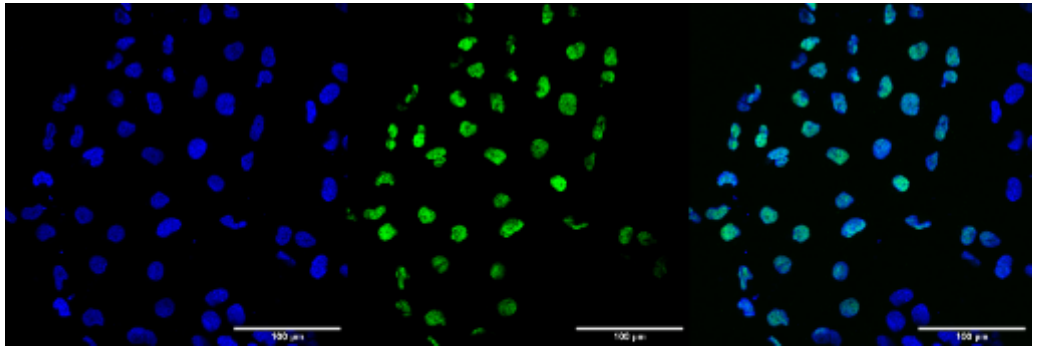

Ectoderm

Hoechst/Nestin/Pax6

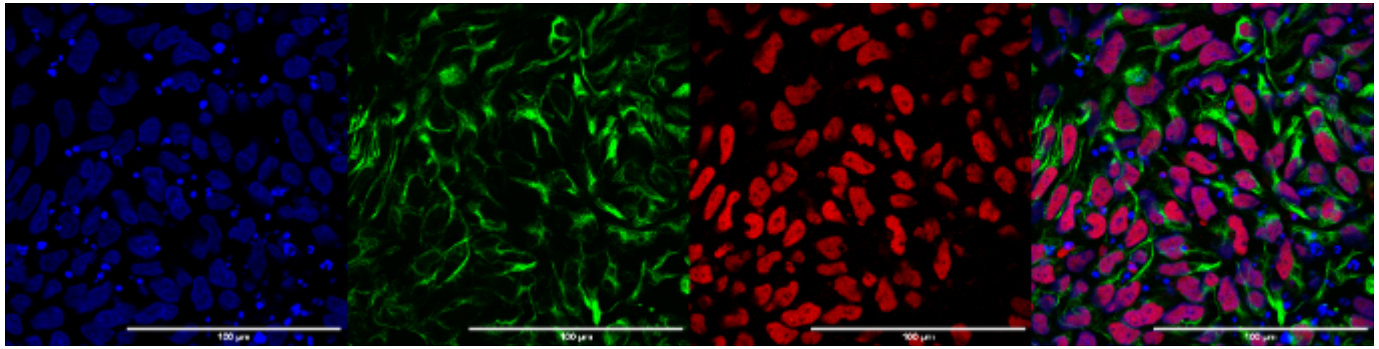

ML 27
Control

Zoomed X2

Endoderm/FoxA2

Hoechst/FoxA2

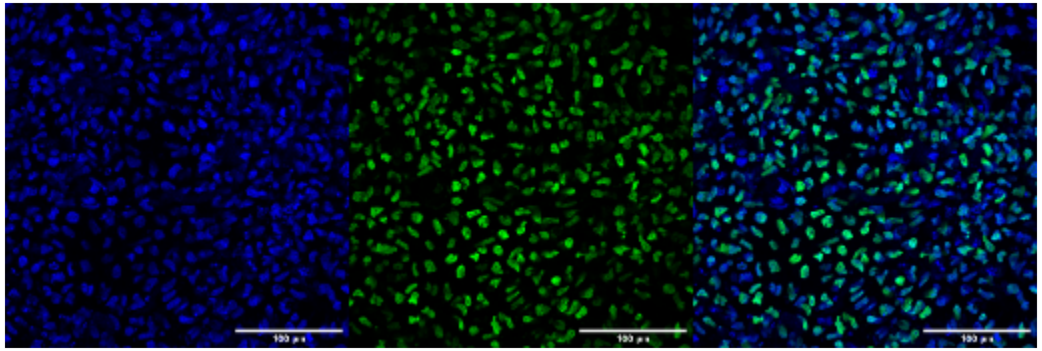

Mesoderm

Hoechst/Brachyury/NCAM

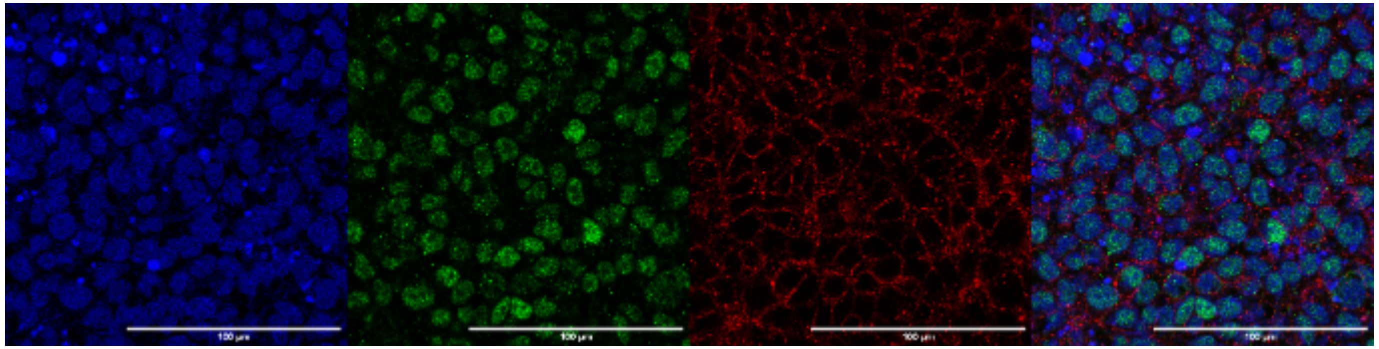

Endoderm/Sox17

Hoechst/Sox17

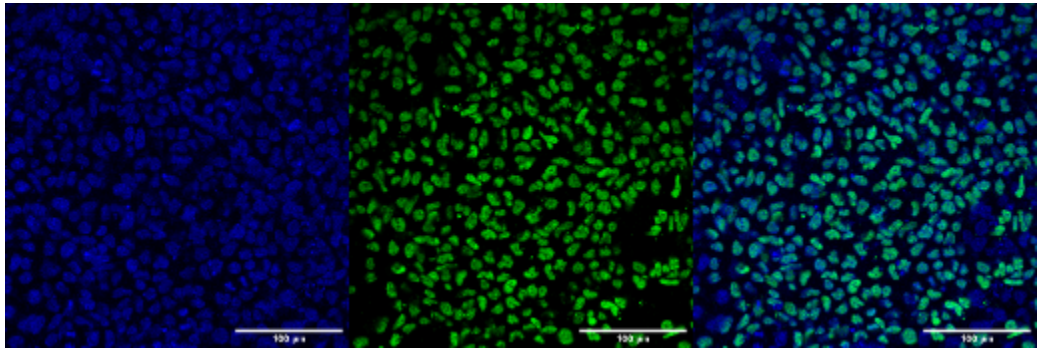

Ectoderm

Hoechst/Pax6/Nestin

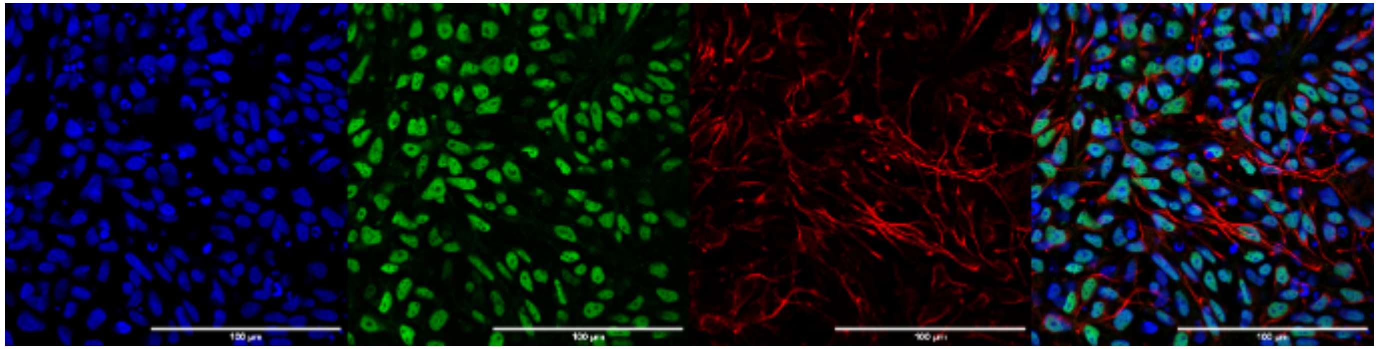

Note: Secondary antibodies for Mesoderm and Ectoderm are switched

ML 300
Control

Zoomed X2

Endoderm/FoxA2

Hoechst/FoxA2

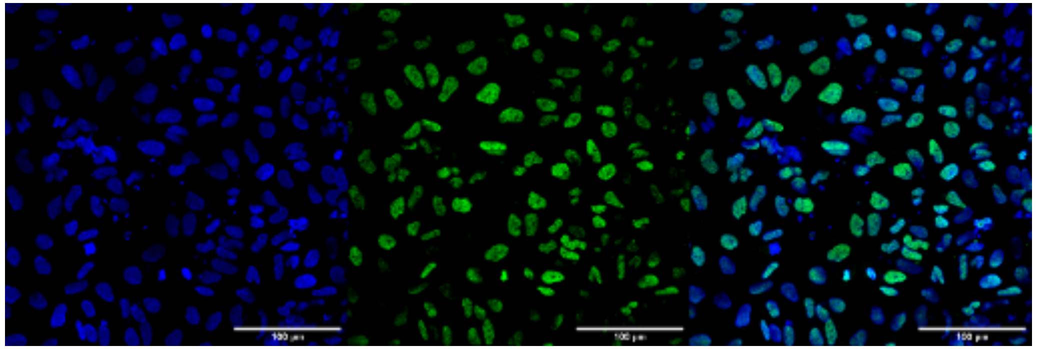

Mesoderm

Hoechst/NCAM/Brachyury

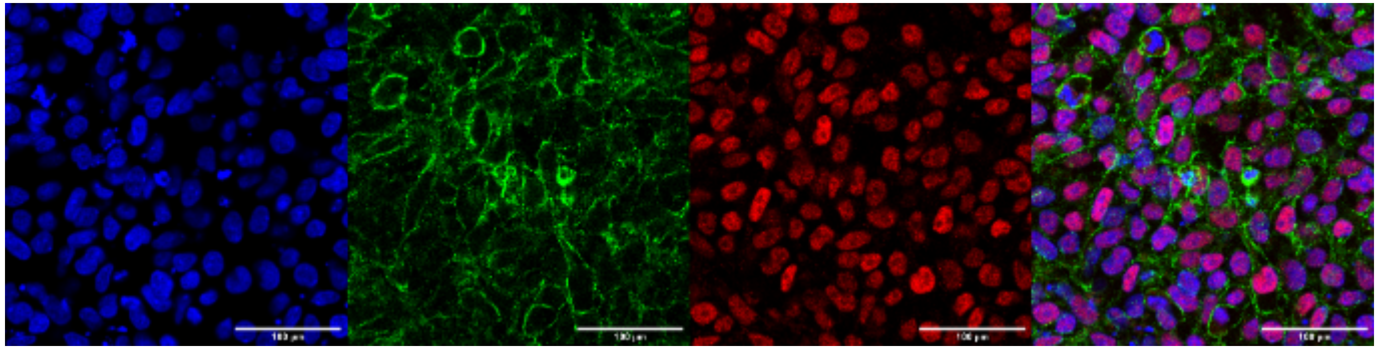

Endoderm/Sox17

Hoechst/Sox17

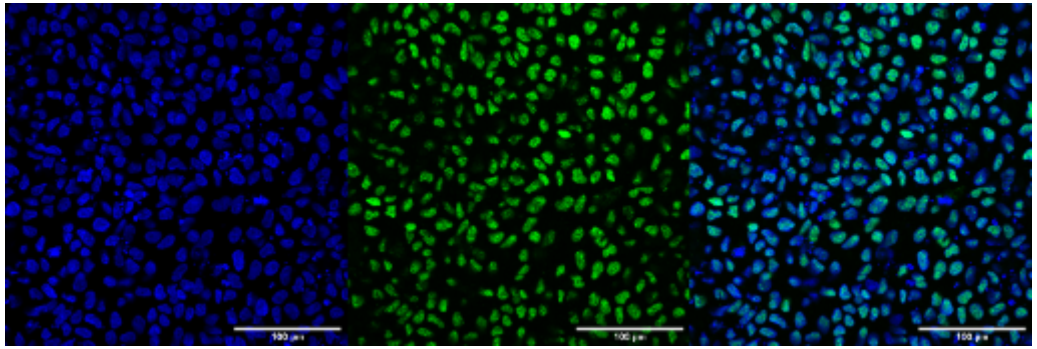

Ectoderm

Hoechst/Nestin/Pax6

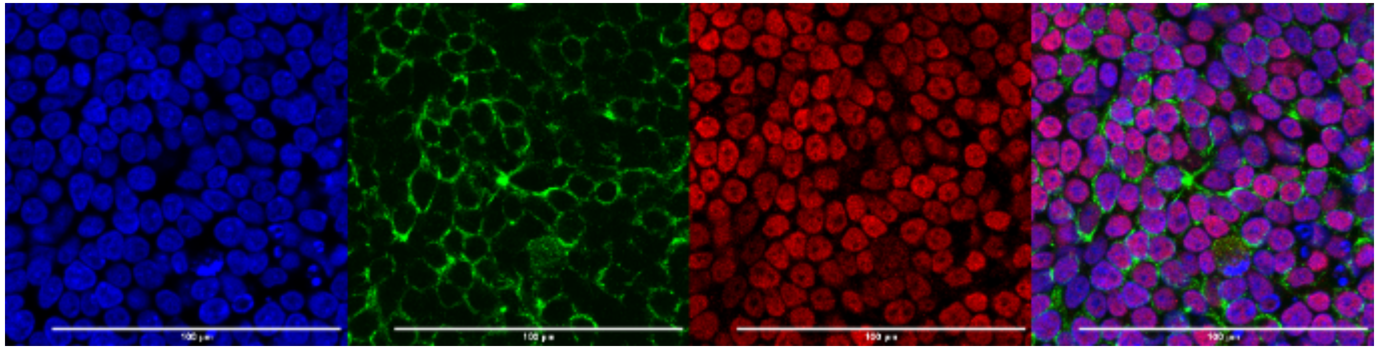

ML 33
SCZ

Zoomed X2

Endoderm/FoxA2

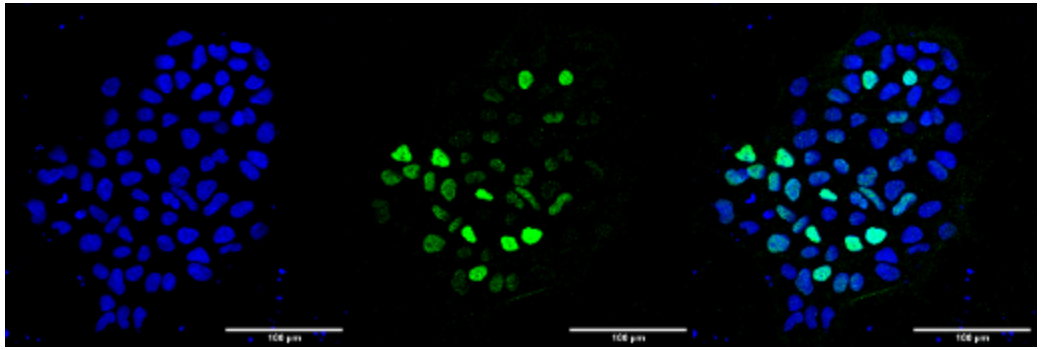

Mesoderm

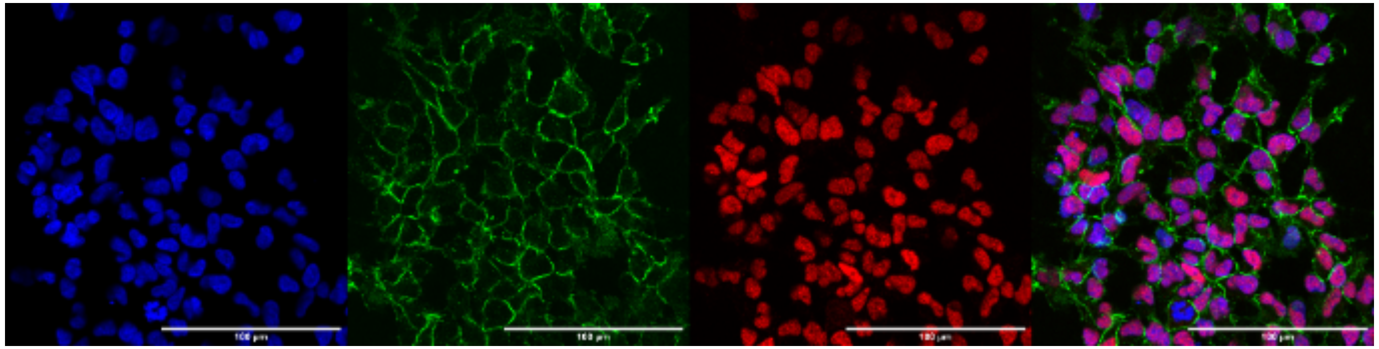

Endoderm/Sox17

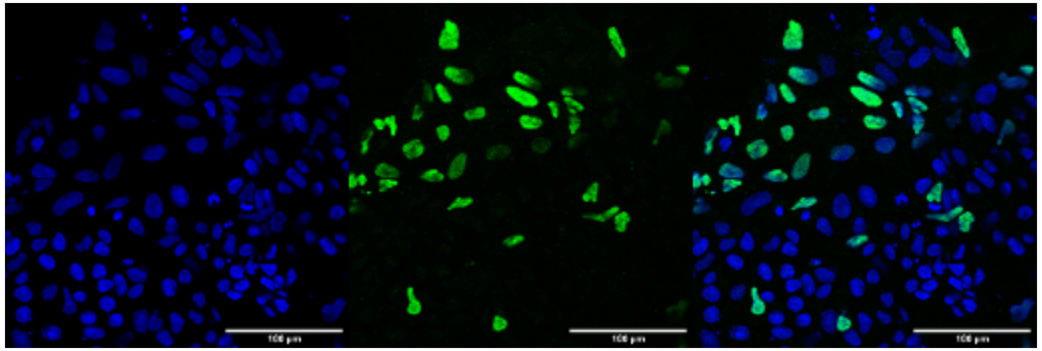

Ectoderm

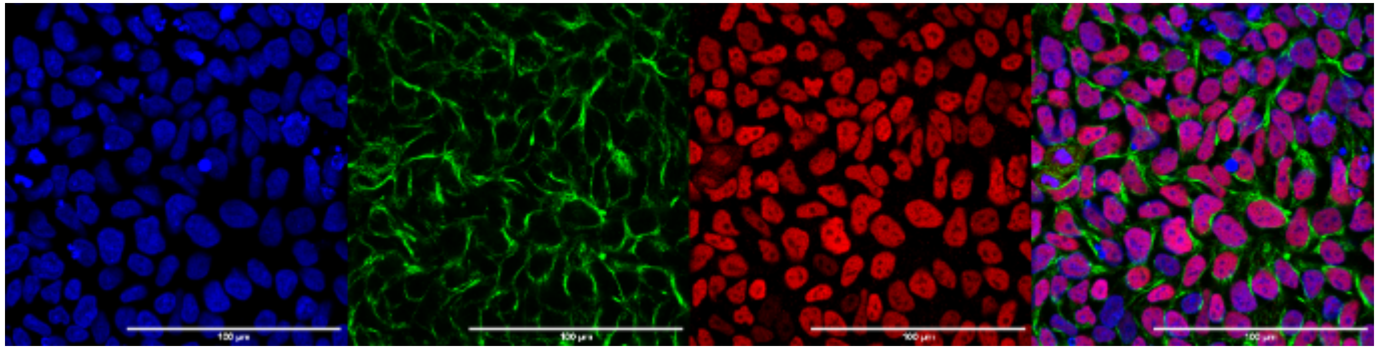

ML 123

SCZ

Endoderm/FoxA2

Hoechst/FoxA2

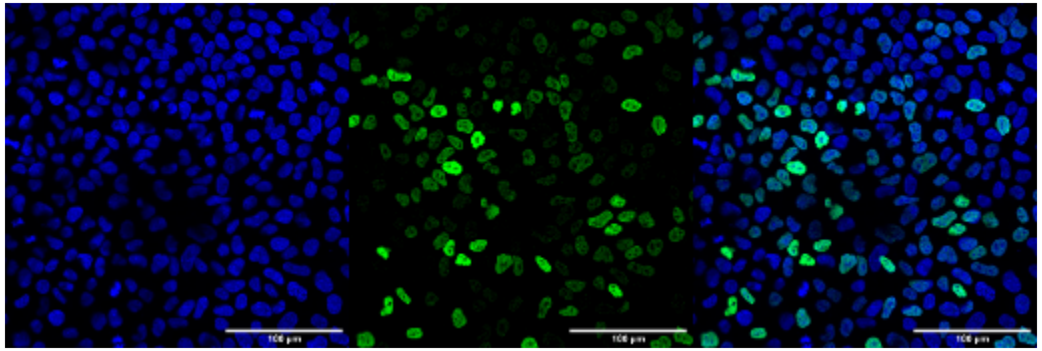

Endoderm/Sox17

Hoechst/Sox17

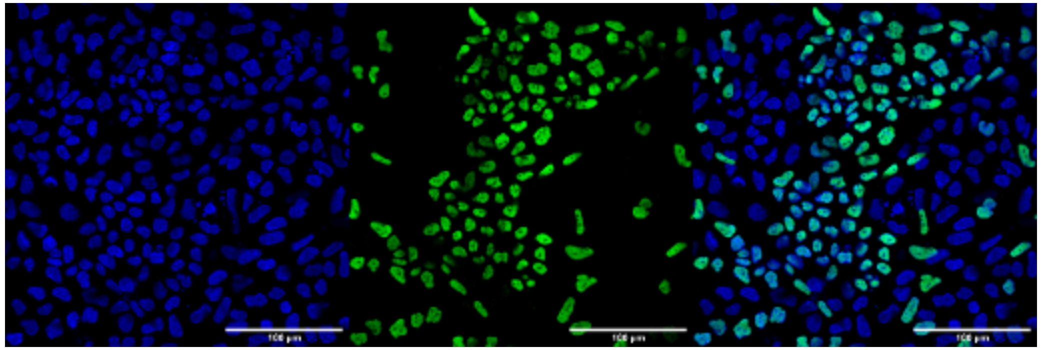

Zoomed X2

Mesoderm

Hoechst/NCAM/Brachyury

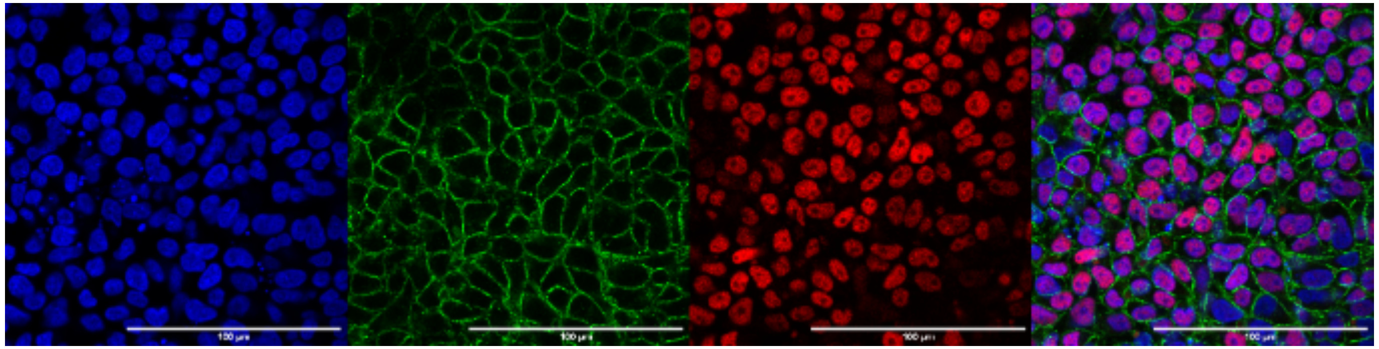

Ectoderm

Hoechst/Nestin/Pax6

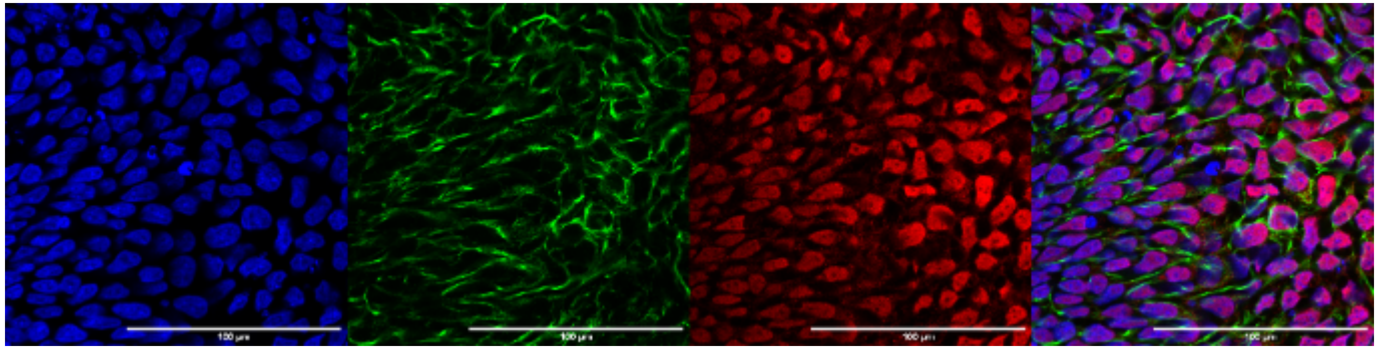

ML 250

SCZ

Zoomed X2

Endoderm/FoxA2

Hoechst/FoxA2

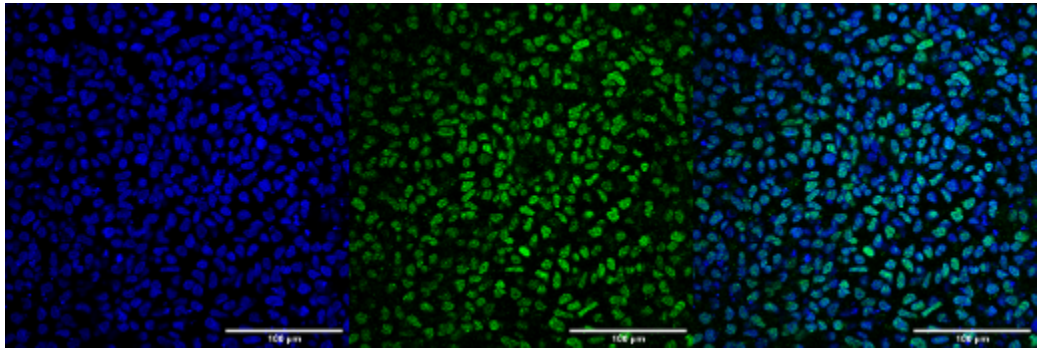

Mesoderm

Hoechst/NCAM/Brachyury

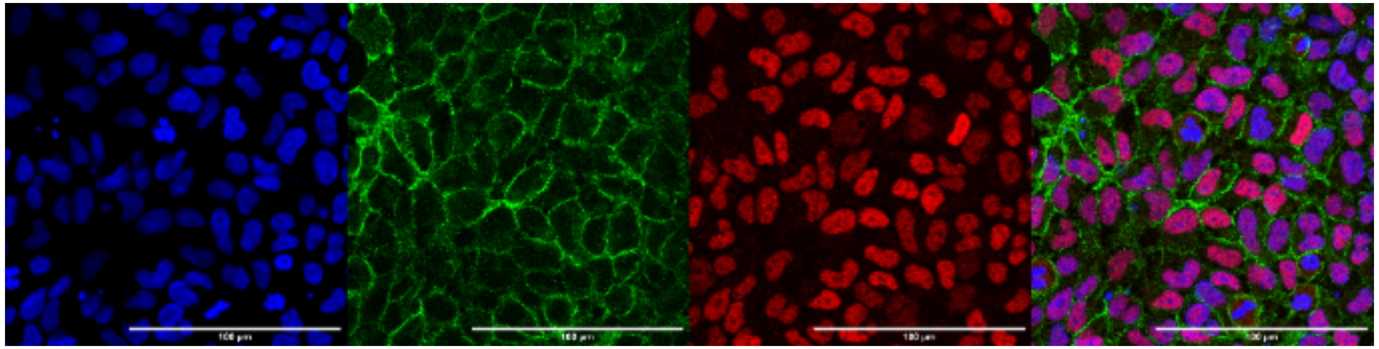

Endoderm/Sox17

Hoechst/Sox17

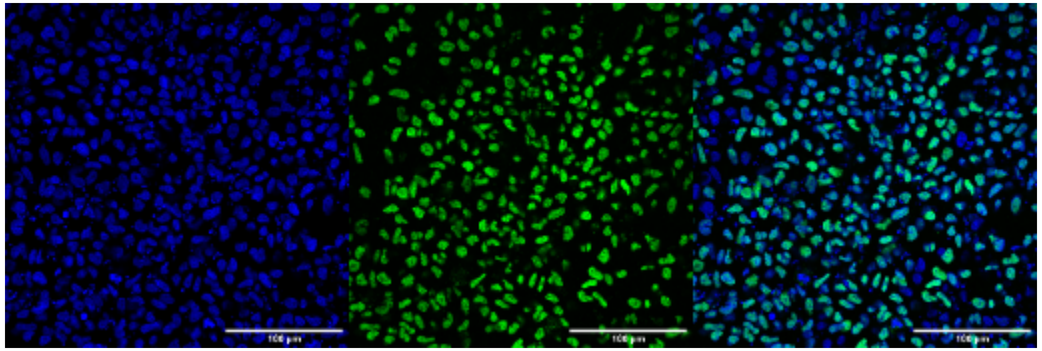

Ectoderm

Hoechst/Nestin/Pax6

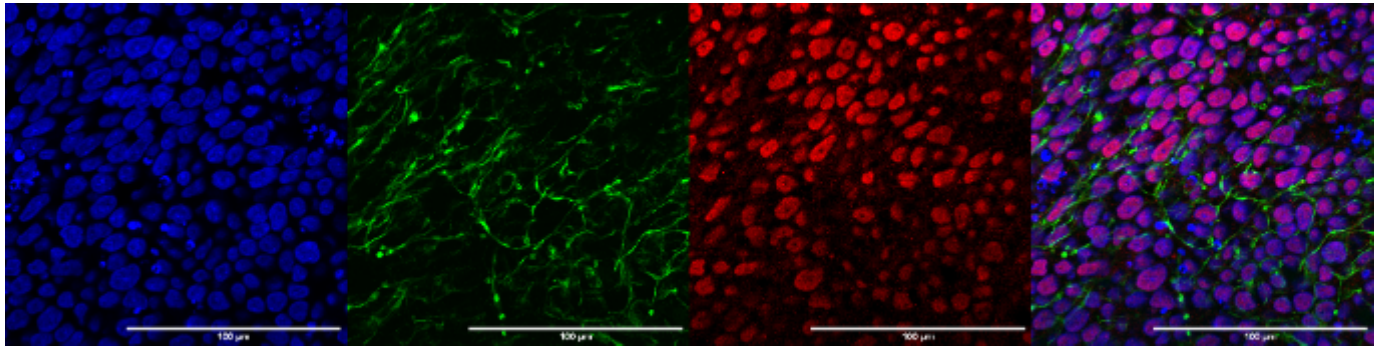

ML 233

SCZ

Endoderm/FoxA2

Hoechst/FoxA2

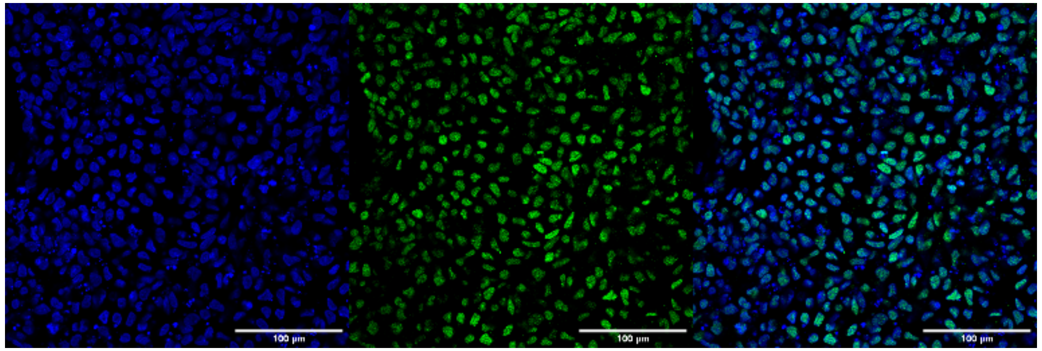

Endoderm/Sox17

Hoechst/Sox17

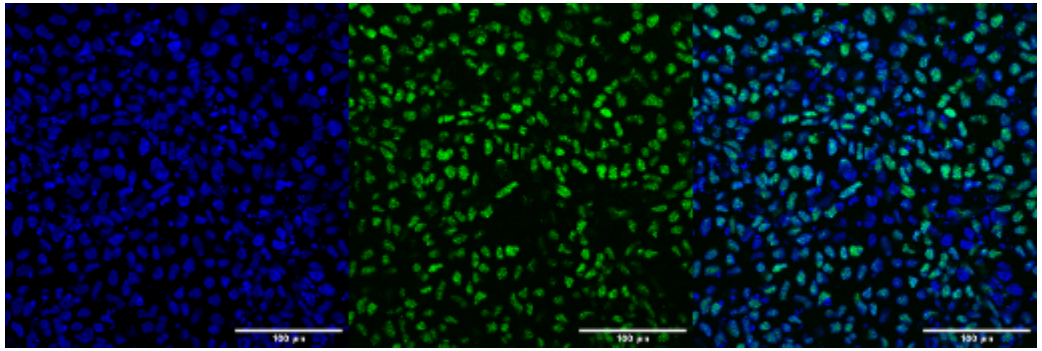

Zoomed X2

Mesoderm

Hoechst/NCAM/Brachyury

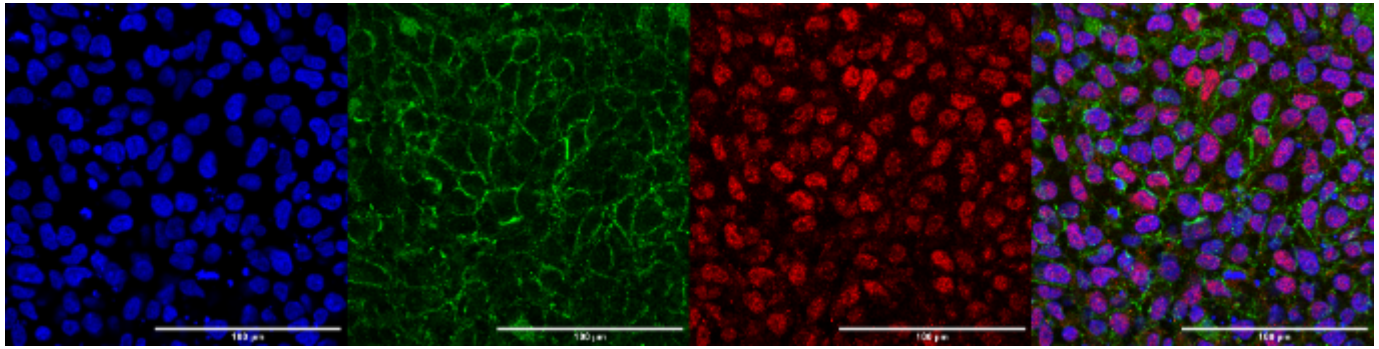

Ectoderm

Hoechst/Nestin/Pax6

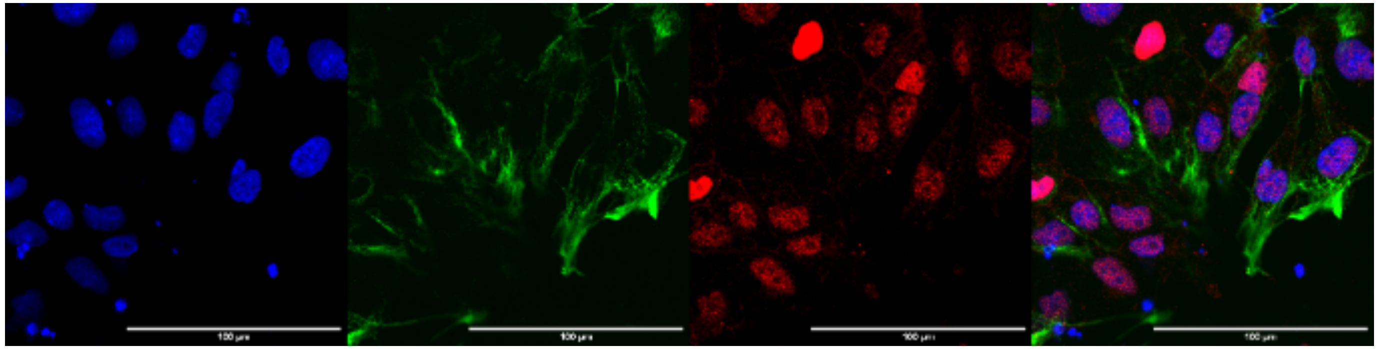

ML 37
SCZ

Zoomed X2

Endoderm/FoxA2

Hoechst/FoxA2

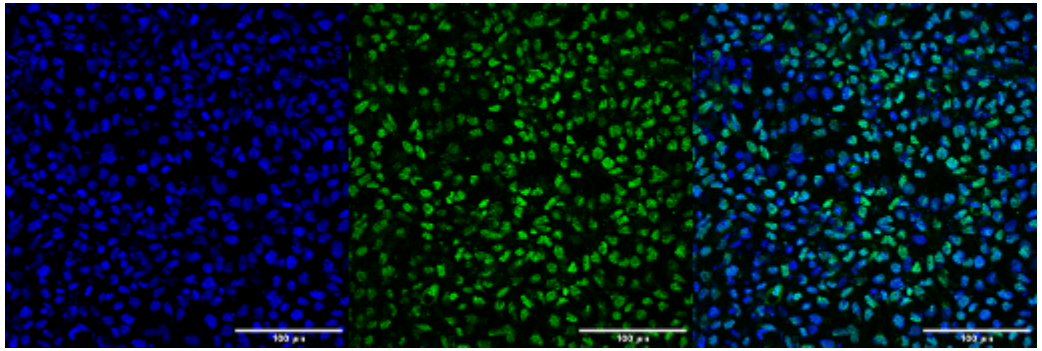

Endoderm/Sox17

Hoechst/Sox17

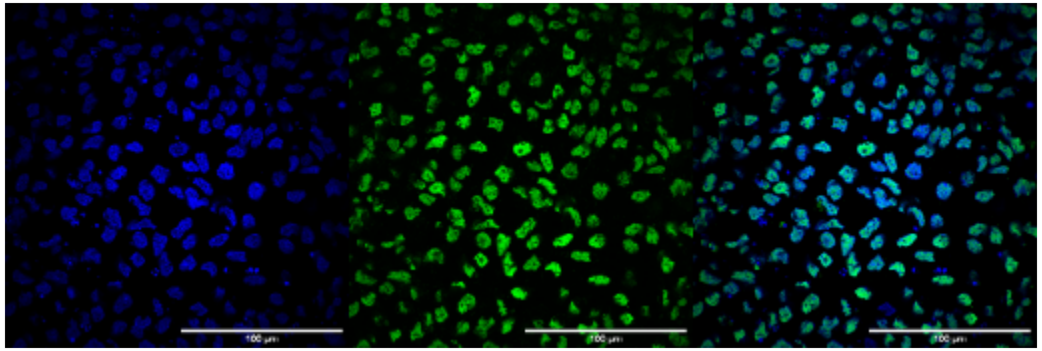

Mesoderm

Hoechst/NCAM/Brachyury

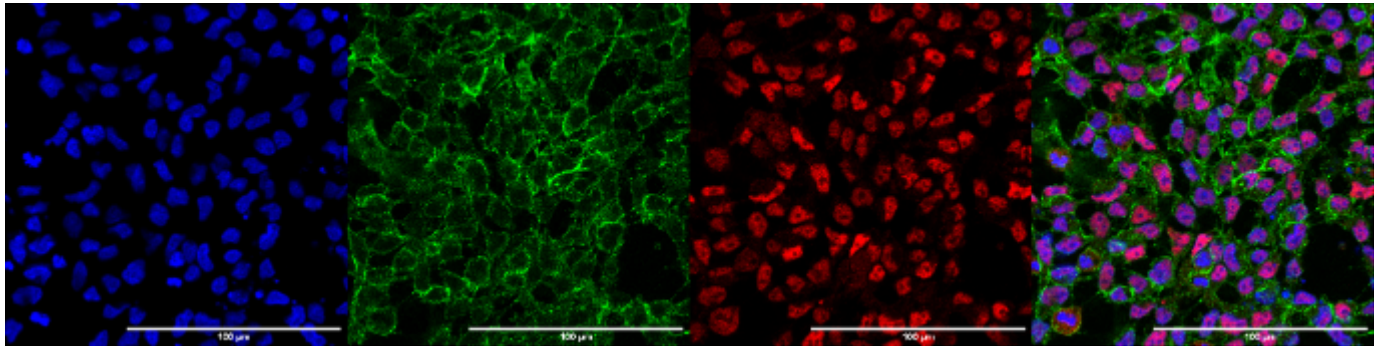

Ectoderm

Hoechst/Nestin/Pax6

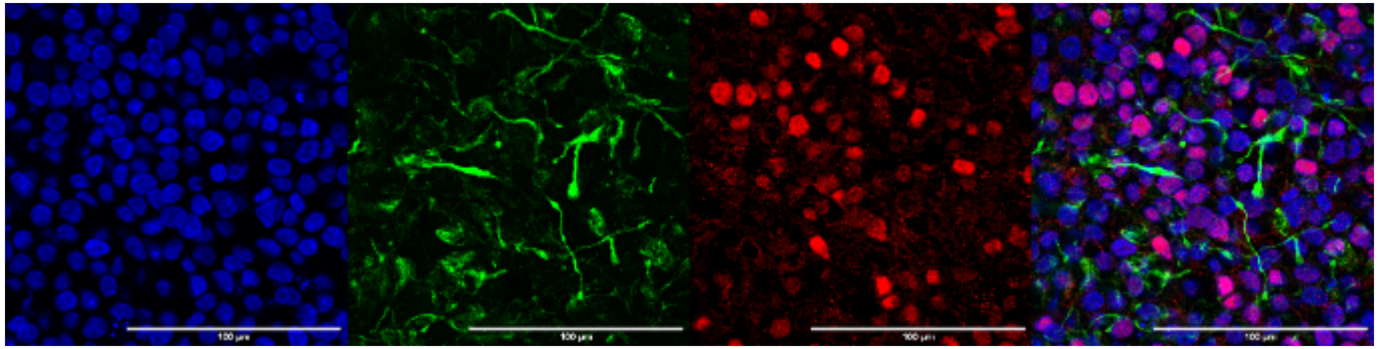

ML 141
SCZ

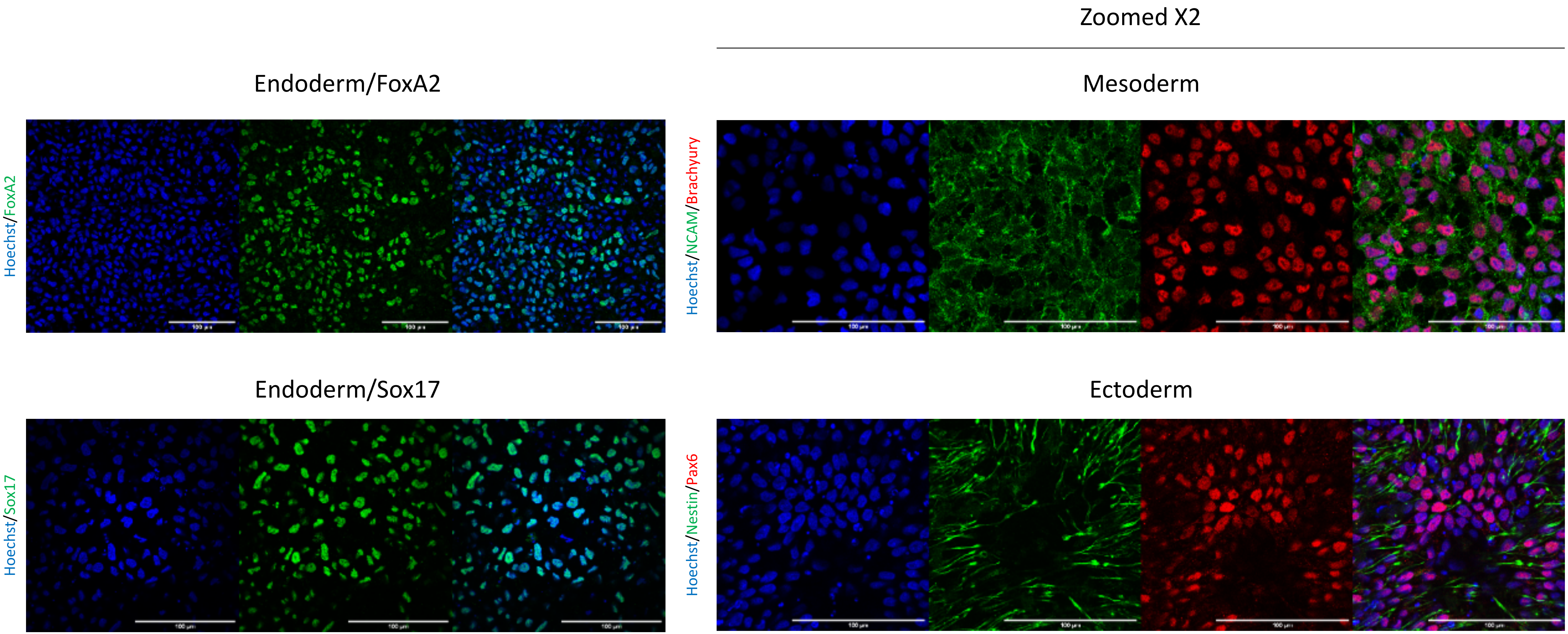
Zoomed X2
Endoderm/FoxA2
Hoechst/FoxA2
Mesoderm
Hoechst/NCAM/Brachyury
Endoderm/Sox17
Hoechst/Sox17
Ectoderm
Hoechst/Nestin/Pax6

ML 164

SCZ

Zoomed X2

Endoderm/FoxA2

Hoechst/FoxA2

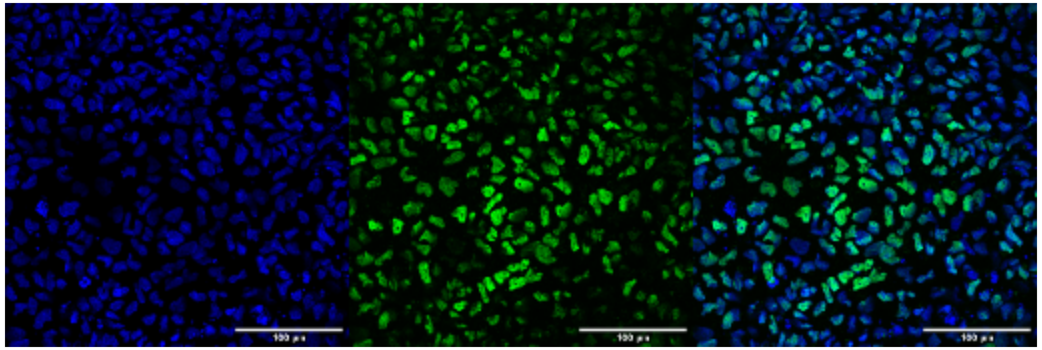

Endoderm/Sox17

Hoechst/Sox17

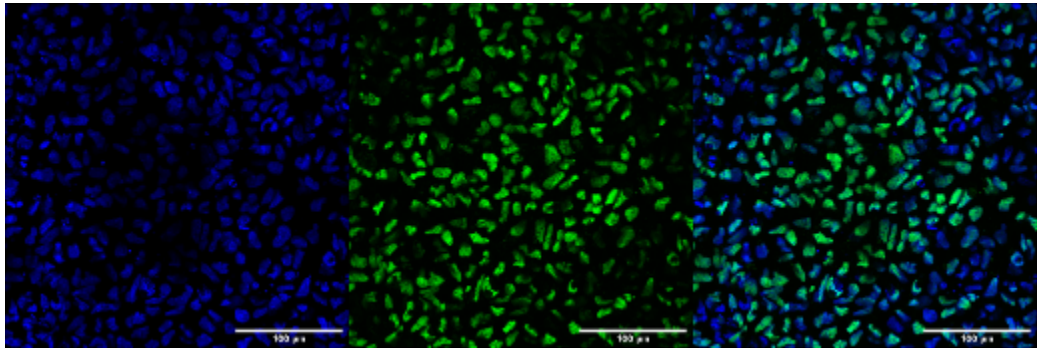

Mesoderm

Hoechst/NCAM/Brachyury

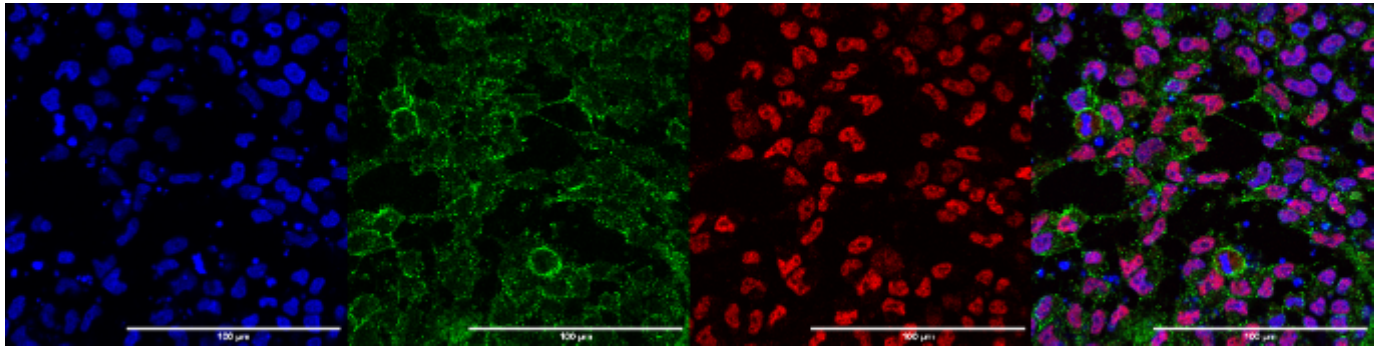

Ectoderm

Hoechst/Nestin/Pax6

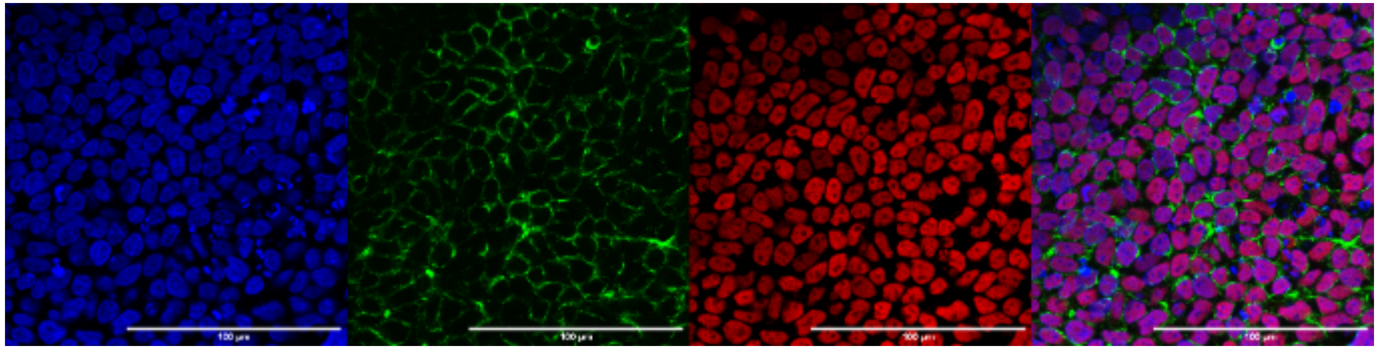

ML 67

SCZ

Endoderm/FoxA2

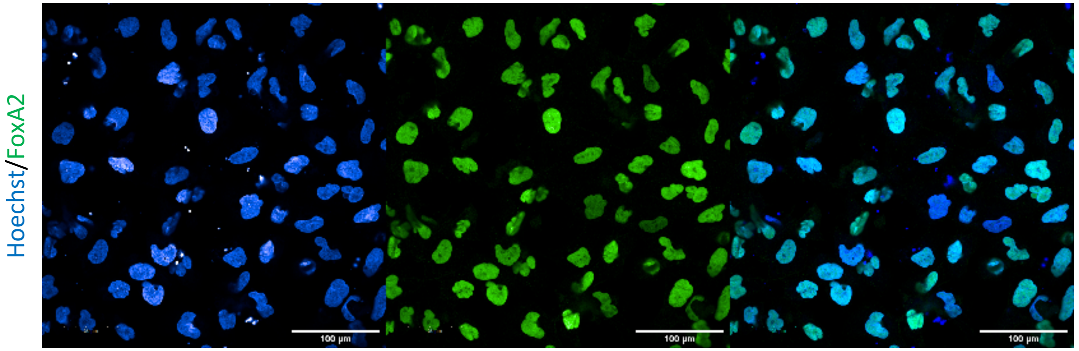

Mesoderm

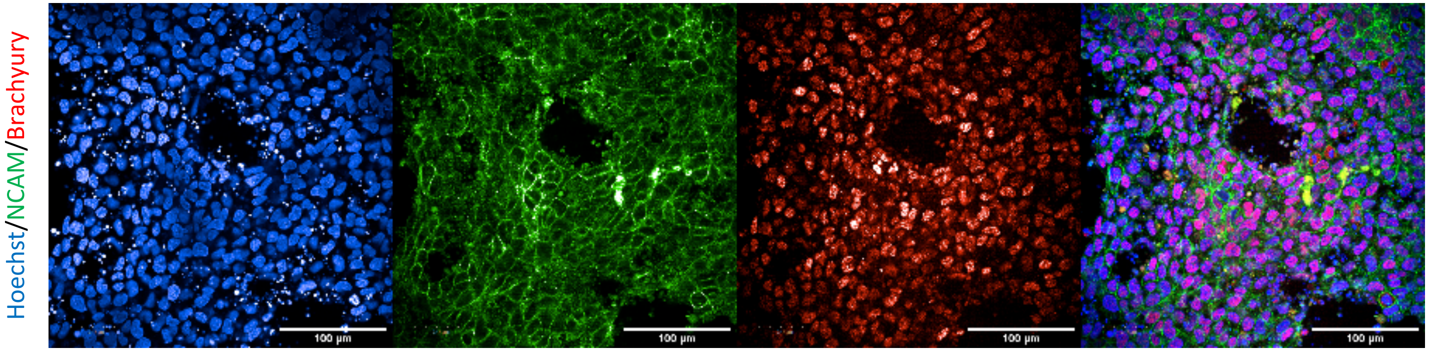

Endoderm/Sox17

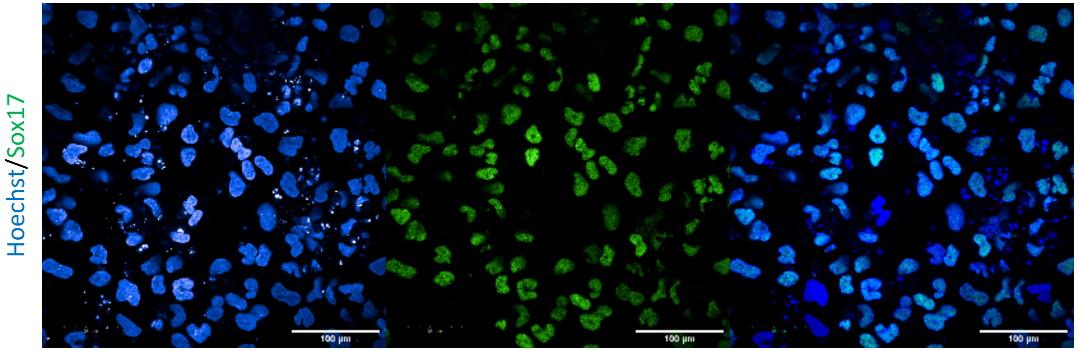

Ectoderm

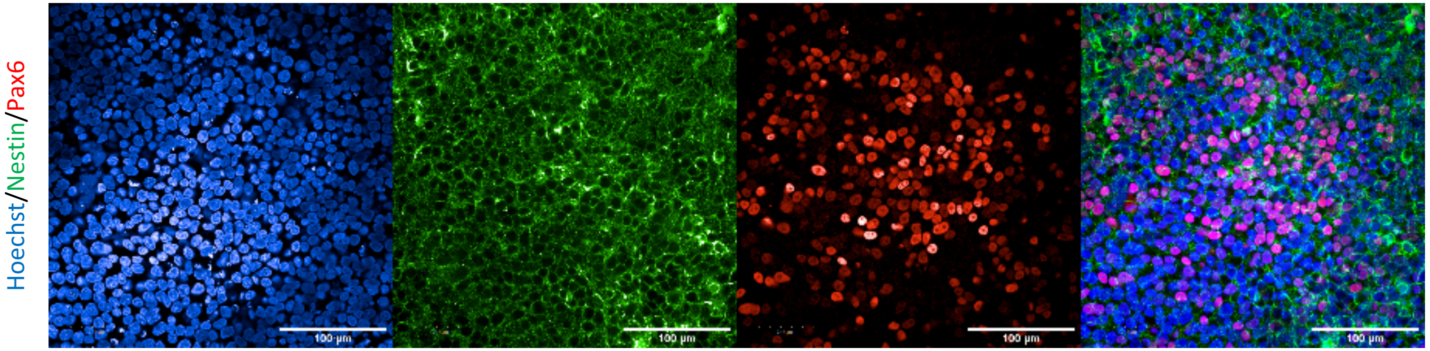

Supplement: Supplementary file 2 — Additional file 2. Data of pluripotency of iPSC lines using trilineage differentiation. [file 13073_2023_1203_MOESM2_ESM.pdf]
